# Methylated DNMT1 and E2F1 are targeted for proteolysis by L3MBTL3 and CRL4^DCAF5^ ubiquitin ligase

**DOI:** 10.1038/s41467-018-04019-9

**Published:** 2018-04-24

**Authors:** Feng Leng, Jiekai Yu, Chunxiao Zhang, Salvador Alejo, Nam Hoang, Hong Sun, Fei Lu, Hui Zhang

**Affiliations:** 1 Department of Chemistry and Biochemistry, University of Nevada, Las Vegas, NV89154 USA; 2 School of Chemical Biology and Biotechnology, Peking University Shenzhen Graduate School, Shenzhen 518055, China

## Abstract

Many non-histone proteins are lysine methylated and a novel function of this modification is to trigger the proteolysis of methylated proteins. Here, we report that the methylated lysine 142 of DNMT1, a major DNA methyltransferase that preserves epigenetic inheritance of DNA methylation patterns during DNA replication, is demethylated by LSD1. A novel methyl-binding protein, L3MBTL3, binds the K142-methylated DNMT1 and recruits a novel CRL4^DCAF5^ ubiquitin ligase to degrade DNMT1. Both LSD1 and PHF20L1 act primarily in S phase to prevent DNMT1 degradation by L3MBTL3-CRL4^DCAF5^. Mouse *L3MBTL3/MBT-1* deletion causes accumulation of DNMT1 protein, increased genomic DNA methylation, and late embryonic lethality. DNMT1 contains a consensus methylation motif shared by many non-histone proteins including E2F1, a key transcription factor for S phase. We show that the methylation-dependent E2F1 degradation is also controlled by L3MBTL3-CRL4^DCAF5^. Our studies elucidate for the first time a novel mechanism by which the stability of many methylated non-histone proteins are regulated.

## Abstract (web summary)

Lysine methylation is increasingly being implicated in the modification of non-histone proteins. Here the authors find that the methylation of DNMT1 and E2F1 are recognized by the protein L3MBTL3 and the ubiquitin E3 ligase CRL4^D^^C^^A^^F5^, which cooperatively target these methylated proteins for ubiquitin-dependent proteolysis.

## Introduction

Lysine-specific methylation is a major protein modification and extensive research has established the key roles of various methylated lysine residues at the amino-terminal regions of histones in modulating chromatin structure and gene expression^1, 2^. Lysine methylations in histones, together with other histone modifications, constitute the “histone code” that acts in a combinatorial or sequential fashion to specify unique downstream function^3^. For example, extensive studies have established that the trimethylation at lysine 4 in histone H3 (H3K4) typically defines a “permissive” chromatin structure that allows active transcription, whereas trimethylations at lysines 9 and 27 in histone H3 (H3K9 and H3K27) are usually associated with repressive chromatin structure that suppresses gene expression^4^. Methylation of lysine 20 in histone H4 (H4K20) plays a critical role in regulating genome stability in response to DNA damage and replication stress^5^. Various mono-, di-, and trimethylated lysine residues in histones usually provide specific protein recognition modules that are “read” by distinct methylation-binding proteins, such as proteins with the Chromo-, Tudor-, MBT-, or PHD-domains, to recruit effector proteins such as transcription factors or chromatin modulators to regulate chromatin organization and gene activities^1, 4, 6, 7^.

Emerging evidence indicates that many non-histone proteins are also methylated at specific lysine residues to regulate their activity or protein stability^8, 9^. The tumor suppressor protein p53 was the first non-histone protein that is found to be monomethylated at lysine 372 (K372) by SET7 (KMT7, SETD7, SET9, or SET7/9), a methyltransferase that was originally identified for its methylation activity toward H3K4 of histone H3^8, 10^. Subsequent reports showed that many non-histone proteins, including DNA (cytosine-5)-methyltransferase 1 (DNMT1), LIN28A, E2F1, NFκB/RelA, ERα, GLI3, RB, FOXO3, TAF10, and STAT3, are also methylated at specific lysine residues by SET7 in embryonic stem cells and various other cells^8, 9, 11–13^. SET7 can also methylate a large number of non-histone proteins at specific lysine residues in vitro^11^, suggesting it may have additional targets in vivo. While the function and regulation of these methylation events in non-histone proteins remain largely uncharacterized, accumulating evidence indicates that methylation of lysine residues by SET7 on a group of non-histone proteins, such as DNMT1, E2F1, NFκB/RelA, FOXO3, and STAT3, triggers the proteolytic destruction of these modified proteins^14–18^.

DNMT1 is a major DNA methyltransferase that methylates cytosine residues in the CpG dinucleotides of the genome^19, 20^. It maintains the CpG DNA methylation patterns in the newly synthesized DNA strands during semi-conservative DNA replication to preserve epigenetic inheritance^19^. DNMT1 is highly regulated during cell cycle progression and is essential for embryonic development, monoallelic expression of genomic imprinted genes, silencing of retrotransposons, X-chromosome inactivation, heterochromatin structure, and tissue specification^20, 21^. Recent studies revealed that the monomethylation of DNMT1 by SET7 at lysine 142 (K142) triggers the destruction of DNMT1 protein^18^. Notably, the methylation of K142 in human DNMT1 by SET7 is prevented by AKT1-mediated phosphorylation of serine 143 (S143)^22^. Similarly, the monomethylation of E2F1, a critical transcription factor that regulates the expression of many genes required for S phase progression, on lysine 185 (K185) by SET7 also triggers the proteolysis of the E2F1 protein, which is abolished when the cells are treated with DNA damage agents such as doxorubicin or etoposide^15^. However, how the methylated lysine residues in these proteins are recognized and processed remains unclear.

The cullin-RING ubiquitin ligases (CRLs) comprise the largest families of ubiquitin E3 ligases^23^. The CRLs employ a large class of substrate-specific receptors to target specifically modified protein substrates for ubiquitin-dependent proteolysis. While many CRL1 ubiquitin ligases, composed of cullin1 (CUL1), SKP1, and one of the F-box proteins, recognize phosphorylated protein substrates, CRL2^VHL^ binds and targets substrates that are marked by proline-hydroxylation for proteolysis^23, 24^. However, the mechanism by which methylation-mediated proteolysis triggered by SET7 remains unclear^25^. We and others have previously shown that the CRL4 core complex, composed of CUL4A (or its paralogue CUL4B), RBX1 (ROC1), and DDB1, employs a specific set of WD40 domain-containing proteins, including the DCAFs, as the substrate-specific subunits to form various CRL4-DCAF ubiquitin E3 ligase complexes in a modular fashion^26–31^. Each of these CRL4-DCAF complexes binds and targets specific protein substrates for ubiquitin-dependent proteolysis. Here, we report that the methylated DNMT1 protein is specifically recognized by a novel methylation-binding protein, which further recruits a unique CRL4 ubiquitin E3 ligase to target DNMT1 for ubiquitin-dependent proteolysis. We also find that the methylation-dependent degradation of E2F1 is controlled by the same mechanism. Our studies thus reveal a novel and common mechanism by which the stability of many methylated non-histone proteins are regulated.

## Results

### LSD1 demethylates the methylated K142 in DNMT1 to stabilize DNMT1

**Fig. 1:**
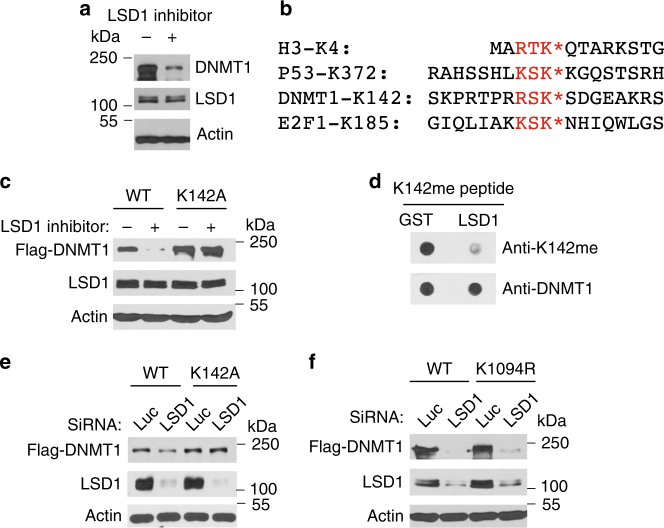
Regulation of DNMT1 proteolysis by LSD1 demethylase. **a** Inhibition of LSD1 leads to downregulation of DNMT1. HCT116 cells were treated with dimethyl sulfoxide (DMSO) or 50 μM LSD1 inhibitor CBB1003 for 12 h and DNMT1 and LSD1 protein levels were analyzed by blotting with indicated antibodies. Actin was used as a loading control. Molecular weight markers are indicated. Experiments were repeated three independent times (biological replicates) with the same conclusion and one example is shown. **b** The conserved lysine residues (K*) methylated by SET7 in a methylation motif with the R/K-S/T-K* consensus sequences in histone H3, p53, DNMT1 and E2F1. **c** The K142A mutant of DNMT1 is resistant to LSD1 inhibition. HCT116 cells were transiently transfected with the Flag-tagged wild-type (WT) or K142A mutant of DNMT expression constructs for 48 h. They were treated with DMSO or 50 μM LSD1 inhibitor as in Fig. 1a. The cells were directly lysed in an SDS-containing lysis buffer and Flag-DNMT1, endogenous LSD1 and actin (loading control) were detected by respective antibodies. **d** LSD1 demethylates the methylated K142 in DNMT1. 1 μg of control GST or GST-LSD1 proteins were incubated with 100 ng of monomethylated K142 or unmethylated peptides for 2 h and the resulting peptides were blotted onto nitrocellulose membrane. The demethylated products were detected by anti-monomethylated K142 antibodies. Total peptides were monitored by anti-DNMT1 antibodies. **e** Loss of LSD1 destabilizes DNMT1 through K142. HCT116 cells were transiently transfected with the Flag-tagged DNMT1 or K142A mutant expression constructs for 12 h. They were then transfected with 50 nM siRNAs of luciferase (Luc, control) or LSD1 for additional 48 h. The cells were processed as in **c**. **f** The K1094R mutant is still sensitive to LSD1 inactivation. HCT116 cells were transiently transfected with the Flag-tagged DNMT1 (WT) or K1094R mutant for 48 h. They were then transfected with 50 nM siRNAs of luciferase (Luc, control) or LSD1 and processed as in **e**. All experiments from **c**, **f** were repeated three independent times with the same conclusion

We have found that treatment of human colorectal carcinoma HCT116 cells with a specific inhibitor for LSD1 (KDM1A)^32, 33^, a demethylase that was originally identified for its ability to remove mono- and dimethylated groups from methylated H3K4^34^, induced rapid downregulation of DNMT1 protein (Fig. 1a). Recent reports indicated that human DNMT1 protein is monomethylated by SET7 at lysine 142 (K142) and this methylation triggers the degradation of DNMT1 protein^18, 22^. In mouse embryonic stem (ES) cells, it was reported that loss of LSD1, which demethylates the lysine 1096 (K1096, equivalent to human K1094), also triggers the proteolysis of the mouse DNMT1 protein^35^. LSD1 also acts as a demethylase to remove the methyl group from the monomethylated K185 in E2F1 to stabilize E2F1 protein^15^. As the R-S-K peptide motif surrounding K142 in DNMT1 resembles the methylation consensus motif, R/K-S/T-K, that is present in many SET7-methylated proteins such as the methylated K4 in histone H3 (H3K4), K372 in p53, and K185 in E2F1 (Fig. 1b)^8, 10, 15, 18, 22^, we tested whether LSD1 acts through the monomethylated K142 in DNMT1 by converting K142 to Alanine (K142A) and examined whether the K142 mutant is still sensitive to LSD1 inhibition. While the ectopically expressed wild-type DNMT1 protein was downregulated after LSD1 was inhibited, the K142A mutant was resistant to LSD1 inhibition (Fig. 1c). To effectively detect the methylated K142 in DNMT1, we developed a specific anti-monomethylated K142 (K142me) peptide antibody for DNMT1 (Supplementary Figure 1A), which only recognized the monomethylated K142 peptide but not the unmethylated cognate peptide. This antibody also detected the increased level of monomethylated K142 in wild-type DNMT1 when SET7 is co-expressed in vivo (Supplementary Figure 1B), which is abolished by the K142A mutation. Our studies also revealed that the recombinant LSD1 can directly and efficiently demethylate the monomethylated K142 peptide in vitro (Fig. 1d). To verify the effect of LSD1 inhibitor, we conducted the siRNA-mediated knockdown of LSD1 in HCT116 cells and found that while the ectopically expressed wild-type DNMT1 protein was downregulated when LSD1 is knocked down, the K142A mutant was resistant to the loss of LSD1 (Fig. 1e). However, we found that conversion of human K1094 to arginine (K1094R) did not significantly stabilize DNMT1 in LSD1 knockdown cells (Fig. 1f). Our studies indicate that LSD1 acts as a demethylase to remove the methyl group from the methylated K142 in DNMT1 to prevent the methylation-dependent proteolysis of DNMT1 in vivo.

### The CRL4 ubiquitin ligase complex is required for DNMT1 degradation

**Fig. 2:**
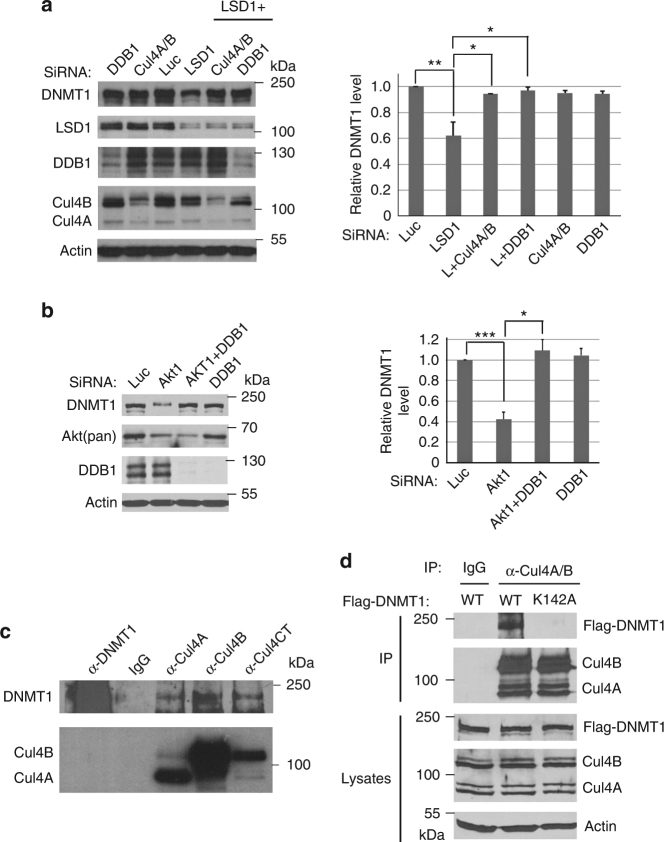
CRL4 regulates the protein stability of DNMT1. **a** Loss of CRL4 core components stabilizes DNMT1. HCT116 cells were transfected with 50 nM siRNAs for each of luciferase, CUL4A + CUL4B (CUL4A/B), and DDB1 for 48 h, in the presence or absence of LSD1 siRNA as indicated. The levels of DNMT1 and CRL4 proteins were analyzed by blotting with indicated antibodies on the left. The protein samples were analyzed with three replicate repeats and the proteins bands were quantified and normalized to the luciferase siRNA control. The quantifications were represented by bar graph with mean and standard deviation (S.D.) for error bars from three replicates. The *P* value of LDS1 to control (Luc) siRNAs was calculated by independent Student’s t-test and the *P* values of double to single knockdowns were calculated by paired Student’s t-test (*P < 0.05, **P < 0.01). Experiments were repeated three independent times with the same conclusion. **b** Loss of DDB1 stabilizes DNMT1 in AKT1 knockdown cells. HCT116 cells were transfected with 50 nM siRNAs for Luc or DDB1, in the presence or absence of AKT1 siRNA as indicated for 48 h. The proteins were analyzed and quantified as in **a**. Student’s *t*-test was used for significant differences. *P < 0.05, ***P < 0.001. **c** The endogenous CRL4 core complexes interact with DNMT1. Endogenous protein complexes were immunoprecipitated (IP) with indicated antibodies and blotted with anti-DNMT1, CUL4A, CUL4B, or CUL4CT (detecting the C termini of CUL4A and CUL4B) antibodies. Non-specific IgG was used as a control. Experiments were repeated four independent times with the same conclusion. **d** CRL4 binding to DNMT1 is abolished by K142A mutation in DNMT1. HCT116 cells were transiently transfected with the Flag-tagged DNMT1 (WT) or K142A mutant constructs for 48 h. The cells were lysed and proteins were immunoprecipitated by anti-CUL4A/CUL4B antibodies or control IgG. The proteins in the CRL4 complexes were detected by anti-Flag and anti-CUL4A/CUL4B antibodies. Experiments were repeated three independent times with the same conclusion

To further investigate how the methylated DNMT1 protein is degraded after LSD1 inactivation, we tried to identify the ubiquitin E3 ligase that mediates this process. Since CRLs represent the largest families of ubiquitin E3 ligases, we used siRNA-mediated knockdown analyses to test the potential involvement of CRLs in DNMT1 degradation. Our initial screen of CRL ubiquitin E3 ligases revealed that downregulation of the components of the CRL4 core complex, consisting of CUL4A or CUL4B, and DDB1, can block the proteolysis of DNMT1 triggered by the loss of LSD1 (Fig. 2a). Treatment of cells with MLN9424, an inhibitor of the neddylation of CRLs, including CRL4, also stabilized DNMT1 in LSD1 knockdown cells (Supplementary Figure 1D). We also found that knockdown of DDB1 blocked the degradation of the methylated DNMT1 protein after the loss of the AKT1 kinase, which prevents SET7-mediated K142 methylation by phosphorylating S143^22^ (Fig. 2b). Since ubiquitin E3 ligases usually directly interact with their protein substrates, we tested and found that the endogenous CUL4A or CUL4B interacts with DNMT1 (Fig. 2c). Our studies also revealed that the interaction of DNMT1 with CUL4A and CUL4B was abolished by the K142A mutation (Fig. 2d), These studies suggest that the CRL4 ubiquitin E3 ligase complex may be involved in the K142 methylation-dependent degradation of DNMT1.

### DCAF5 serves as a specific subunit of CRL4 for DNMT1 degradation

**Fig. 3:**
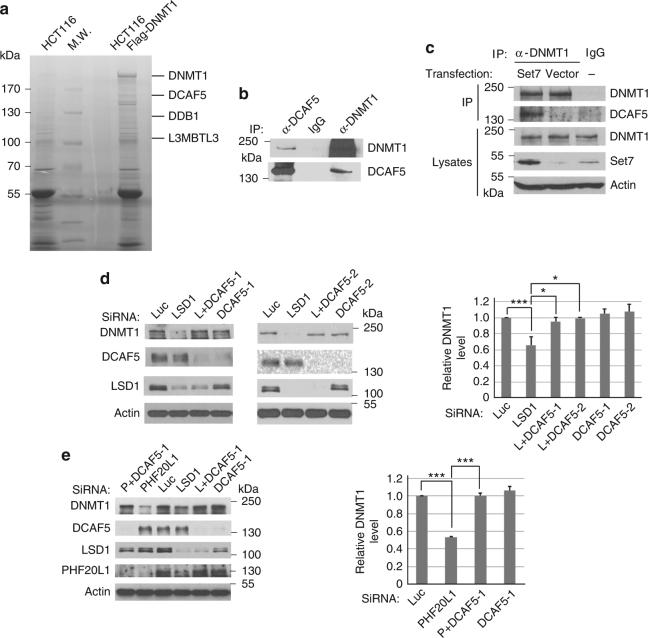
Regulation of DNMT1 protein stability by DCAF5. **a** Isolated DNMT1 protein complexes contain DDB1, DCAF5, and L3MBTL3 proteins (see Supplementary Table 1 for additional information). The Flag-knock-in DNMT1 protein complexes were isolated from HCT116-Flag-DNMT1 cells by anti-Flag M2 antibody affinity resins or from control HCT116 cells without the Flag-DNMT1. The proteins associated with the isolated Flag-DNMT1 complexes were separated in protein gel, excised, trypsinized, and derivative peptides were fractionated by nano-liter liquid chromatography and identified by mass spectrometry. The positions of some proteins associated with the DNMT1 complex are shown. MW: molecular weight markers. **b** Endogenous DCAF5 and DNMT1 proteins interact with one another. DCAF5 and DNMT1 from HCT116 cells were immunoprecipitated and detected by western blot analyses as indicated. Experiments were repeated three independent times with the same conclusion. **c** The interaction between DNMT1 and DCAF5 is enhanced by SET7 expression. The SET7 expressing or empty control vectors were transfected into HCT116 cells for 48 h, treated with proteasome inhibitor MG132 (5 μg/ml) for last 6 h, and interactions between DNMT1 and DCAF5 were analyzed by co-immunoprecipitation and western blotting analyses. **d** Downregulation of DCAF5 prevents DNMT1 degradation triggered by LSD1 deficiency. HCT116 cells were transfected with 50 nM of luciferase siRNA and two independent DCAF5 siRNAs in the presence or absence of LSD1 (LSD1 or L) siRNA. The protein levels of DNMT1, DCAF5, actin, and LSD1 were examined in total cell lysates by western blotting using indicated antibodies and quantified. The *P* value of LSD1 to control (Luc) siRNAs was calculated by independent Student’s t-test and the *P* values of double to single knockdowns were calculated by paired Student’s *t*-test. *P < 0.05, ***P < 0.001. **e** HCT116 cells were transfected with 50 nM of luciferase and DCAF5 siRNAs in the presence or absence of PHF20L1 or LSD1 siRNAs for 48 h. The protein levels of DNMT1, DCAF5, actin, LSD1 and PHF20L1 were examined and quantified. Student’s *t*-test was calculated for significant differences. ***P < 0.001. Quantifications for all experiments were represented by bar graph with mean and S.D. for error bars from three replicates, as described in **a**

The CRL4 ubiquitin E3 ligases usually use one of substrate-specific subunit proteins, such as DCAFs, to interact with specific protein substrates to target them for degradation^26–31^. To further identify the substrate-specific subunit protein of CRL4 for DNMT1, we tried to isolate the DNMT1 protein complex and search for additional CRL4 components. We used a HCT116 cell line in which the C terminus of one of the *DNMT1* alleles is fused with the Flag-epitope-tag (Flag-DNMT1 knock-in) to facilitate the purification of the endogenous DNMT1 protein complexes by anti-Flag antibody affinity chromatography^36^ (Fig. 3a). The proteins associated with the purified DNMT1 complexes were identified by an Orbitrap XL mass spectrometry system^28, 37^. While a large number of DNMT1-derived peptides were recovered, we repeatedly identified a small number of peptides derived from CUL4B, DDB1 and DCAF5, a CRL4-associated DCAF with unknown function, in the DNMT1 protein complexes (Fig. 3a and Supplementary Table 1).

Since the CRL4 core complex is involved in DNMT1 degradation (Fig. 2), we tested whether DCAF5 is indeed associated with the DNMT1 complexes. Our independent immuno-co-precipitation analyses confirmed the interactions between the endogenous DNMT1 and DCAF5 proteins (Fig. 3b and Supplementary Figure 1E). We also functionally tested the involvement of DCAF5 in DNMT1 degradation. While reduction of LSD1 triggers the proteolysis of DNMT1, co-knockdown of DCAF5 using two independent DCAF5 siRNAs and LSD1 siRNA consistently prevented the degradation of endogenous DNMT1 protein (Fig. 3d). Since it was reported that the K142-methylated DNMT1 is protected by the binding of PHF20L1^38^, an MBT-Tudor domain-containing protein encoded by a putative oncogene that is amplified or over-expressed in aggressive breast cancers^39^, we knocked down PHF20L1 and found there is increased degradation of DNMT1, and this degradation is also prevented by co-knockdown of DCAF5 (Fig. 3e). Our subsequent analysis showed that the interaction between DNMT1 and DCAF5 is greatly enhanced when SET7 is ectopically expressed (Fig. 3c). These studies revealed that CRL4^DCAF5^ is likely involved in the methylation-dependent degradation of DNMT1 catalyzed by SET7.

### L3MBTL3 is involved in DNMT1 degradation

In histones, methylated lysine residues are usually recognized by distinct methylation-binding proteins with the Chromo-, Tudor-, MBT-, and PHD-domains^1, 4, 6, 7^. During the purification of DNMT1 protein complexes, we have also noticed the presence of peptides derived from L3MBTL3 (Fig. 3a and Supplementary Table 1), a protein that contains the three tandem repeated malignant brain tumor (MBT) domain. L3MBTL3 was previously isolated as an accessory component of Polycomb Repressive Complex 1 (PRC1)^40, 41^. *L3MBTL3* is mutated in medulloblastoma and is further implicated in other pathological disorders such as multiple sclerosis, insulin resistance, prostate cancer and breast cancer^42–46^. Other MBT domain-containing proteins, such as L3MBTL1 and L3MBTL2, were reported to bind to mono- or dimethylated lysine residues in histones to induce chromatin compaction or transcriptional repression^6, 46^. Although the function of L3MBTL3 remains unknown, mouse null mutation of *L3MBTL3* causes late embryonic lethality^47^.

**Fig. 4:**
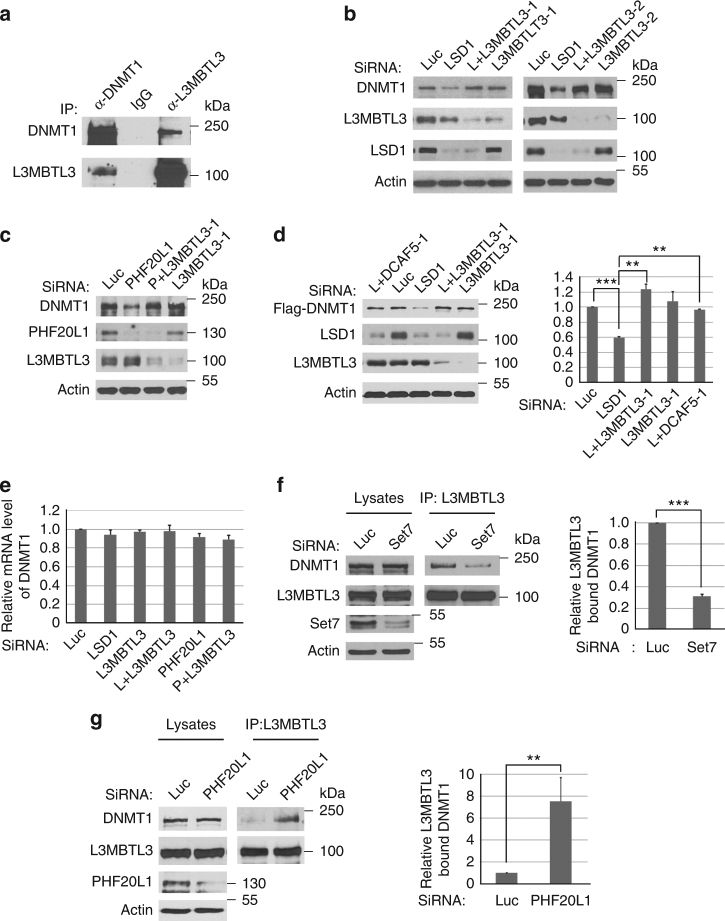
L3MBTL3 regulates DNMT1 protein stability. **a** Interaction between DNMT1 and L3MBTL3. DNMT1 and L3MBTL3 were immunoprecipitated from HCT116 cells and western blotted with their respective antibodies. **b** Loss of L3MBTL3 stabilizes DNMT1 in LSD1 deficient cells. HCT116 cells were transfected with luciferase and two independent L3MBTL3 siRNAs in the presence or absence of LSD1 siRNA for 48 h. Proteins were analyzed by indicated antibodies. **c** Downregulation of L3MBTL3 prevents DNMT1 degradation in PHF20L1 knockdown cells. Experiments were conducted as in Fig. 4b except PHF20L1 siRNA is used. **d** Loss of DCAF5 or L3MBTL3 stabilizes Flag-DNMT1 in LSD1 deficient cells. The Flag-DNMT1 in pMSCV-Puro was stably expressed in 293 cells and its protein stability was analyzed after transfecting either L3MBTL3 or DCAF5 siRNAs, together with or without LSD1 siRNA as in Fig. 2a, using Student’s t-test for significant differences. **P < 0.01 and ***P < 0.001. **e** Loss of L3MBTL3 does not significantly affect DNMT1 mRNA levels. The DNMT1 mRNA levels were measured in triplicated samples in cells treated with siRNAs for Luciferase, LSD1, PHF20L1, L3MBTL3, LSD1+L3MBTL3, and PHF20L1+L3MBTL3 by RT–qPCR. The quantifications are represented by bar graph with mean and standard deviation (S.D.) for error bars from three replicate samples and normalized to luciferase siRNA control. **f** Downregulation of SET7 reduces the interaction between DNMT1 and L3MBTL3. HCT116 cells were transfected with control and SET7 siRNAs and the interaction between DNMT1 and L3MBTL3 was analyzed by immunoprecipitation and western blotting. Significant differences was calculated by Student’s *t*-test, as in Fig. 2a. ***P < 0.001. **g** Downregulation of PHF20L1 promotes the interaction between DNMT1 and L3MBTL3. HCT116 cells were transfected with control and PHF20L1 siRNAs for 48 h. The proteasome inhibitor (5 μg/ml) MG132 was added for last 6 h to stabilize DNMT1 for analyzing and quantifying the levels of the interaction between DNMT1 and L3MBTL3. Student’s *t*-test was calculated, as in Fig. 2a, for significant differences. **P < 0.01. Quantifications for all experiments were represented by bar graph with mean and S.D. for error bars from three replicates, as described in Fig. 2a. All experiments were repeated three independent times with the same conclusion

Since CRL4^DCAF5^ does not appear to contain a recognizable methylation-binding domain, we tested whether L3MBTL3 is involved in DNMT1 degradation (Fig. 4). Our independent verification confirmed that endogenous DNMT1 and L3MBTL3 proteins indeed reciprocally associated with one another (Fig. 4a). In addition, co-silencing of L3MBTL3 using two independent siRNAs, together with LSD1 or PHF20L1 siRNAs, showed that downregulation of L3MBTL3 stabilized endogenous DNMT1 protein in LSD1 or PHF20L1 deficient cells (Fig. 4b, c, Supplementary Figure 2A, 2B). To rule out the potential regulation of DNMT1 by L3MBTL3 at the transcriptional level, we ectopically and stably expressed a Flag-tagged DNMT1 protein under the retroviral LTR promoter control in 293 cells and found that downregulation of LSD1 also destabilized the ectopically expressed Flag-DNMT1 protein, but the degradation of Flag-DNMT1 is prevented by the knockdown of L3MBTL3 or DCAF5 (Fig. 4d), indicating that L3MBTL3 and DCAF5 regulate DNMT1 at the protein level. Our examination of the mRNA levels of DNMT1 by the reverse transcriptional quantitative PCR (RT–qPCR) analyses also indicated that loss of L3MBTL3 did not significantly affect DNMT1 transcription (Fig. 4e). These studies suggest that L3MBTL3 is specifically and functionally required for DNMT1 proteolysis triggered by LSD1 or PHF20L1 deficiency.

### L3MBTL3 specifically interacts with the monomethylated K142 in DNMT1

**Fig. 5:**
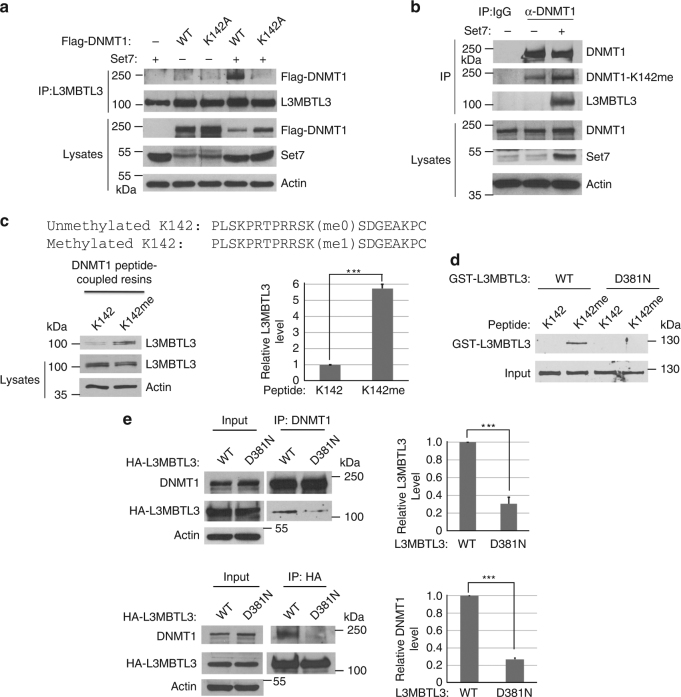
L3MBTL3 interacts with the methylated K142 to regulate the stability of the methylated DNMT1 protein. **a** The interaction between L3MBTL3 and DNMT1 is dependent on SET7 and K142. The Flag-WT-DNMT1 and its K142A mutant and SET7 constructs were co-transfected into HCT116 cells. The interactions of DNMT1 and the mutant with L3MBTL3 were analyzed by anti-L3MBTL3 immunoprecipitation and western blotting as indicated. **b** SET7 stimulated the methylation of K142 and interaction between DNMT1 and L3MBTL3. SET7 expression constructs were transfected into HCT116 cells. The interactions between DNMT1 and L3MBTL3 and the methylated DNMT1 were analyzed by immunoprecipitation and western blotting with anti-methylated K142 and other indicated antibodies. **c** L3MBTL3 preferentially binds to the monomethylated K142 peptide. Sulfolink-immobilized DNMT1 peptides containing the monomethylated (Kme1) or cognate unmethylated K142 (Kme0) were incubated with 250 μg of HCT116 extracts for 4 h. L3MBTL3 binding to the peptide resins was analyzed by anti-L3MBTL3 Western blotting and quantified with independent Student’s t-test. ***P < 0.001. **d** L3MBTL3 directly interacts with the monomethylated K142 peptide. The K142me0 and Kme1 peptide resins were pre-incubated with 5 μg of GST for 2 h to reduce the background. The resins were then incubated with 1.5 μg of GST-L3MBTL3 or its D381N mutant for 4 h at 4 °C as indicated. The washed resins were blotted with anti-L3MBTL3 antibodies. **e** Mutation of the MBT domain in L3MBTL3 reduces its binding to DNMT1 in vivo. HA-tagged L3MBTL3 (WT) and the D381N mutant expression constructs were transfected into HCT116 cells for 42 h and then treated with MG-132 for additional 6 h. The interaction between DNMT1 and the wild-type or mutant HA-L3MBTL3 proteins were examined by reciprocal immunoprecipiation and western blotting with anti-DNMT1 or anti-HA antibodies. Student’s *t*-test was calculated for significant differences. ***P < 0.001. All quantifications were represented by bar graph with mean and S.D. for error bars from three replicates and all experiments were repeated three independent times with the same conclusion

Since L3MBTL3 contains the MBT repeated domain that is considered as a “reader” of various mono- or dimethylated lysine residues^6, 46^, we examined whether the interaction between DNMT1 and L3MBTL3 is affected by the methylated K142 status. We found that downregulation of SET7, which monomethylates K142, significantly reduced the interaction between DNMT1 and L3MBTL3 (Fig. 4f), whereas knockdown of PHF20L1 (in the presence of MG132, an inhibitor of the 26S proteasome, for 6 final hours), which binds the methylated K142 and protects methylated DNMT1 from degradation, greatly enhanced their association (Fig. 4g). In addition, while expression of SET7 stimulated the interaction between L3MBL3 and DNMT1, the K142A mutant cannot interact with L3MBTL3, even in the presence of SET7 (Fig. 5a). The expression of SET7 specifically increased the level of monomethylated K142 in DNMT1, which can be detected by our anti-monomethylated K142 antibody (Fig. 5b). We found that this increased methylation in K142 promoted the interaction between DNMT1 and L3MBTL3 (Fig. 5a, b). However, these SET7-dependent methylation processes were abolished by the K142A mutation (Supplementary Figure 1B, Fig. 5a, b). These studies suggest that the interaction between L3MBTL3 and DNMT1 involves the methylation of K142 by SET7.

To determine whether L3MBTL3 specifically binds to the monomethylated K142 in DNMT1, we analyzed the affinity of L3MBTL3 in cell lysates toward a pair of DNMT1 peptides (24 amino acid residues and an extra cysteine at the C terminus) that contain either a monomethylated K142 or a cognate non-methylated K142 control peptide immobilized by covalent disulfide linkage to Sepharose resins through the C-terminal cysteine residue at the end of the peptides^48^ (Fig. 5c). Our analysis revealed that endogenous L3MBTL3 protein from HCT116 soluble cell lysates preferentially binds to the monomethylated K142 peptide (Fig. 5c). To determine whether L3MBTL3 can directly bind to the methylated K142 peptide, we constructed a recombinant fusion protein between glutathione-S-transferase (GST) and L3MBTL3. We found that the purified recombinant GST-L3MBTL3 protein directly and preferentially binds to the monomethylated K142 peptide in vitro (Fig. 5d), while other MBT domain containing proteins, such as GST-L3MBTL1 and GST-L3MBTL2, showed no significant bindings, as compared with that of GST protein control, to the unmethylated or monomethylated peptide resins (Supplementary Figure 2C). The MBT domain of L3MBTL3 contains three tandem MBT repeats and previous studies suggest that the second MBT repeat usually plays a critical role in L3MBTL3 or related MBT domain-containing proteins for binding to the methylated peptides^6^. We tested the MBT domain of L3MBTL3 in their binding to the methylated K142 peptide by converting Aspartate 381 to Asparagine (D381N), a critical amino acid residue in the second MBT repeat of L3MBTL3 for methylated lysine binding^6^. As shown in Fig. 5d, the GST-D381N mutant form of L3MBTL3 failed to interact with the monomethylated K142 peptide.

We also expressed the HA-tagged wild-type L3MBTL3 and the D381N mutant by transfecting into cells and analyzed their interaction with DNMT1. Our studies revealed that the D381N mutant of L3MBTL3 greatly reduced its affinity to bind DNMT1 (Fig. 5e). Our studies thus strongly indicate that L3MBTL3 directly and specifically recognizes and binds to the monomethylated K142 in DNMT1 through its MBT domain.

### L3MBTL3 interacts with DCAF5 to target DNMT1 for polyubiquitination

**Fig. 6:**
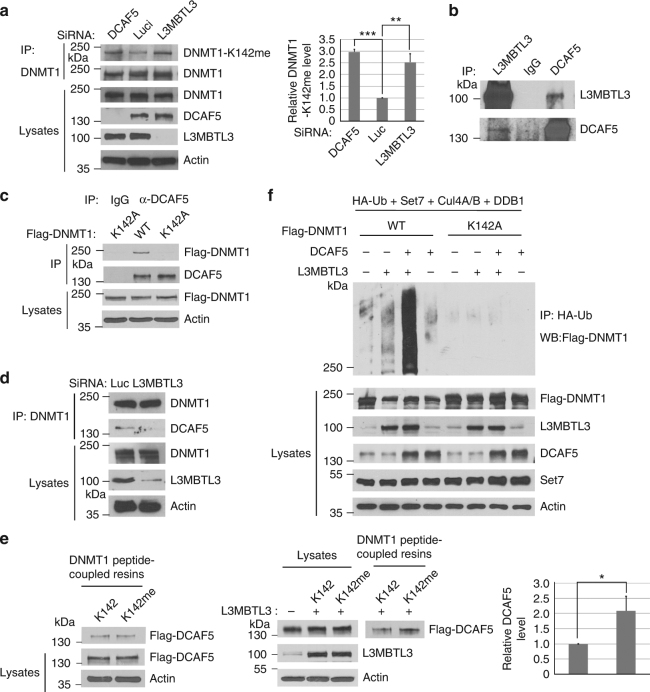
L3MBTL3 interacts with CRL4^DCAF5^ and recruits DCAF5 onto the methylated DNMT1 protein. **a** Downregulation of DCAF5 or L3MBTL3 increases the K142-methylated DNMT1. HCT116 cells were transfected with 50 nM of control, DCAF5 and L3MBTL3 siRNAs. DNMT1 was immunoprecipitated by anti-DNMT1 antibody and western blotted by anti-methylated K142 or anti-DNMT1 antibodies. Student’s *t*-test was caculated for significant differences. **P < 0.01 and ***P < 0.001. **b** L3MBTL3 and DCAF5 interact. Endogenous DCAF5 and L3MBTL3 proteins were immunoprecipitated from HCT116 cells and western blotted with indicated antibodies. **c** The interaction between DCAF5 and DNMT1 is abolished by K142 mutation. Flag-WT-DNMT1 and its K142A mutant constructs were transfected into HCT116 cells and the interactions between DCAF5 and Flag-DNMT1 or the K142A mutant were analyzed by anti-DCAF5 immunoprecipitation and western blotting with anti-Flag and DCAF5 antibodies. **d** Downregulation of L3MBTL3 abolished the interaction between DNMT1 and DCAF5. Endogenous DNMT1 and DCAF5 proteins were immunoprecipitated by anti-DNMT1 antibodies from control and L3MBTL3 siRNA-mediated knockdown cells (42 h of siRNA and MG132 for additional 6 h). The immunoprecipitated proteins were blotted with anti-DCAF5 and DNMT1 antibodies. **e** L3MBTL3 is required for preferential binding of DCAF5 to the monomethylated K142 peptide. HCT116 extracts expressing a stable Flag-DCAF5 or Flag-DCAF5 and L3MBTL3 were prepared. 250 μg extract proteins were incubated with the monomethylated K142 or the cognate unmethylated peptide resins as in Fig. 5c. The binding of Flag-DCAF5 to the peptide resins with or without L3MBTL3 were examined by anti-Flag and L3MBTL3 antibodies. Student’s *t*-test was used for significant differences. *P < 0.05. **f** L3MBTL3 and CRL4^DCAF5^ target DNMT1 for polyubiquitination in vivo. Flag-WT DNMT1 and Flag-K142 A mutant expressing constructs were co-transfected into 293 cells together with vectors expressing HA-tagged ubiquitin (HA-Ub), SET7, CUL4A, CUL4B, and DDB1, in the presence or absence of L3MBTL3 and DCAF5 expressing constructs as indicated. Proteins were immunoprecipitated with anti-HA antibodies and western blotted with the anti-Flag-DNMT1 antibodies as indicated. Quantifications for all experiments were represented by bar graph with mean and S.D. for error bars from three replicates and Student’s t-test was used for significant differences, as described in Fig. 2a

Since L3MBTL3 is a protein that “reads” the methylated K142 in DNMT1 (Figs. 4 and 5) and since CRL4^DCAF5^ also regulates the proteolysis of DNMT1 (Figs. 2 and 3), L3MBTL3 may act as a methylation binding protein to recruit the CRL4^DCAF5^ ubiquitin ligase to target the methylated DNMT1 protein for proteolysis through its interaction with CRL4^DCAF5^. Several lines of evidence support this model. We found that downregulation of either DCAF5 or L3MBTL3 led to the accumulation of the monomethylated DNMT1 (Fig. 6a). The interactions between DNMT1 and DCAF5 or L3MBTL3 were stimulated by SET7 but abolished by the K142A mutation (Figs. 3c, 5a, b and 6c). Endogenous L3MBTL3 indeed interacted with the endogenous DCAF5 and DDB1 proteins (Fig. 6b, Supplementary Figure 1F). Furthermore, downregulation of L3MBTL3 greatly reduced the interaction between DCAF5 and DNMT1 (Fig. 6d). Our studies also revealed that while it is often difficult to detect the preferential binding of DCAF5 to the methylated K142 peptide beads, expression of L3MBTL3 promotes the preferential binding of DCAF5 to the monomethylated K142 peptide (Fig. 6e).

**Fig. 7:**
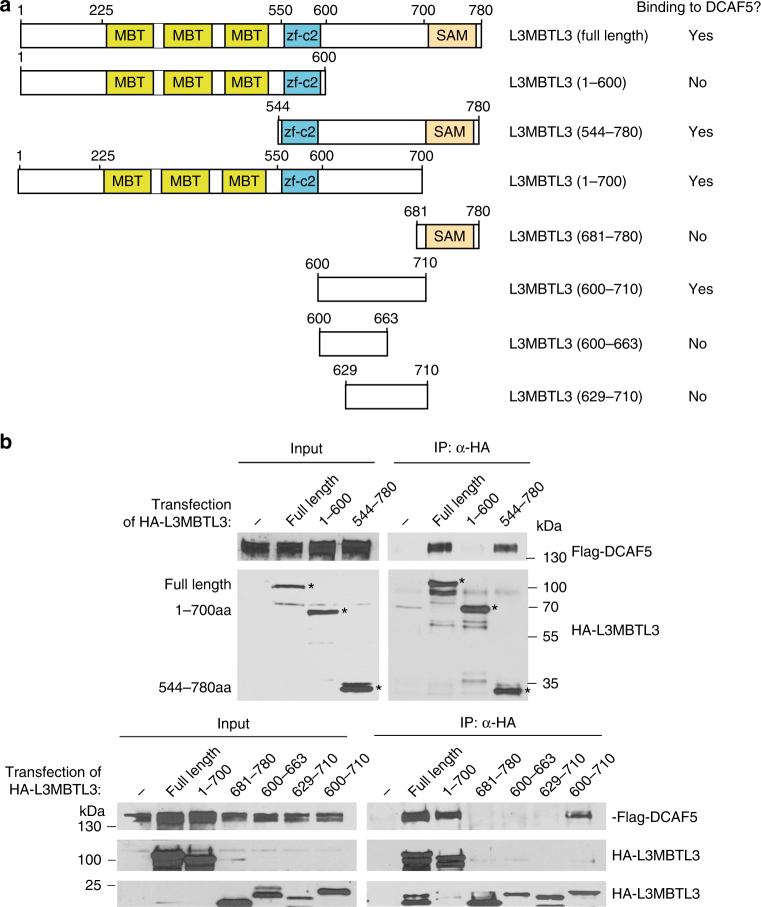
L3MBTL3 interacts with DCAF5 with a unique region between the zf-c2 and the SAM domains. **a** Schematic illustration of the domains of the L3MBTL3 protein, various L3MBTL3 mutants, and their ability to interact with DCAF5, as shown in **b**. **b** The region (600-710) between the zf-c2 and SAM domains of L3MBTL3 interacts with DCAF5. HCT116 cells were transiently transfected with the expression constructs of the HA-tagged L3MBTL3 wild-type (WT, full length 1-780) or various HA-tagged mutants of L3MBTL3, together with the Flag-tagged DCAF5 expressing construct for 48 h. The cells were lysed and WT and mutant L3MBTL3 proteins were immunoprecipitated by anti-HA antibodies and the presence of Flag-DCAF5, HA-L3MBTL3 and mutant proteins were detected by western blotting with anti-Flag and anti-HA antibodies. The input cell lysates show the expression of Flag-DCAF5, HA-L3MBTL3 and mutant proteins in transfected cells. Experiments were repeated four independent times with the same conclusion and one example is shown

We also characterized the interaction between DCAF5 and L3MBTL3. L3MBTL3 contains three conserved domains: the MBT repeats, a conserved zf-C2 domain with unknown function, and a SAM (Sterile Alpha Motif) domain for oligomerization of SAM domain-containing proteins^49^ (Fig. 7a). We found that while the full length L3MBTL3 (780 amino acid residues) interacts with DCAF5 (Fig. 7a, b and Supplementary Figure 3A), the region between 610–700 amino acid residues in L3MBTL3 is both necessary and sufficient to interact with DCAF5. Our analysis also revealed that the carboxyl-terminal region of DCAF5 is required for the interaction with L3MBTL3, independent of the amino-terminal WD40 repeated domain of DCAF5^29^ (Supplementary Figure 3B). Thus, our studies indicate that L3MBTL3 and DCAF5 interact with each other through specific protein regions in these two proteins.

Consistent with the interaction between L3MBTL3 and DCAF5, our studies further showed that while expression of L3MBTL3 or DCAF5 can induce weak polyubiquitination of DNMT1 in 293 cells, co-expression of DCAF5 and L3MBTL3, in the presence of CRL4 core complex and SET7, greatly stimulated the polyubiquitination reaction of DNMT1 protein and this effect was abolished by K142A mutation in DNMT1 (Fig. 6f). These studies demonstrate that L3MBTL3 recruits the CRL4^DCAF5^ ubiquitin E3 ligase complex to interact with the methylated K142 in DNMT1 and consequently targets the DNMT1 protein for polyubiquitination-dependent proteolysis.

### The methylation-dependent DNMT1 proteolysis is cell cycle regulated

**Fig. 8:**
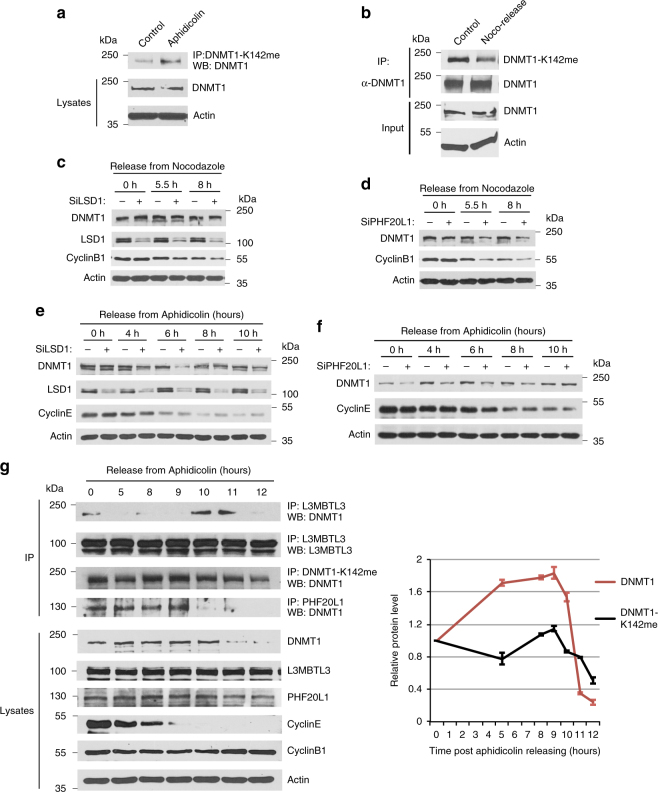
The methylation-dependent proteolysis of DNMT1 is cell cycle regulated. **a** S phase cells contain higher levels of methylated DNMT1. HCT116 cells were treated with DMSO or 5 μg/ml aphidicolin for 18 h. Methylated DNMT1 was immunoprecipitated with anti-monomethylated K142 antibodies and blotted with anti-DNMT1 antibodies. **b** G1 cells are low in methylated DNMT1. HCT116 cells were treated with DMSO or 40 ng/ml nocodazole for 14 h. The mitotic round-up cells were collected, washed, and re-plated in fresh medium. The G1 cells were lysed at 7.5 h after re-plating. DNMT1 proteins were immunoprecipitated by anti-DNMT1 antibodies and blotted by anti-monomethylated K142 and anti-DNMT1 antibodies. **c** HCT116 cells were transfected with luciferase and LSD1 siRNAs for 30 h, treated with nocodazole for 14 h, and mitotic rounded-up cells were collected and released into fresh medium, as in **b**. The late mitotic, early and mid G1 cells were collected at 0, 5.5, and 8 h. DNMT1, LSD1, cyclin B1 (degraded in G1), and actin were examined by western blotting. **d** The same as **c** except that HCT116 cells were treated with PHF20L1 siRNAs for 30 h, followed by mitotic shake-off and release into G1. **e** HCT116 cells were transfected with luciferase and LSD1 siRNAs for 30 h and synchronized at the G1/S border by sequential treatment of 2.5 mM thymidine and 5 μg/ml aphidicolin. The released S phase cells were collected at indicated time points. The levels of DNMT1, LSD1, cyclin E (high in G1/S and early S) and actin were analyzed by western blotting. **f** The same as **e** except that HCT116 cells were treated with PHF20L1 siRNAs, **g** HCT116 cells were synchronized at the G1/S border as in **e**. Cells were collected at various indicated time points after release and the levels of the K142-methylated DNMT1, total DNMT1, and their interactions with L3MBTL3 or PHF20L1 were analyzed by immunoprecipitation and western blotting. Quantifications were represented by bar graph with mean and standard deviation (S.D.) for error bars from three replicates and experiments were repeated three independent times with the same conclusion

DNMT1 normally maintains the epigenetic inheritance of DNA methylation patterns by methylating cytosine residues in the CpG dinucleotides of the genome on the newly replicated DNA strands in S phase^18, 22^. It also interacts with other regulatory or epigenetic modulatory proteins independent of its DNA methyltransferase activity^50–54^. We found that the methylation levels of the DNMT1 protein are higher in S phase cells arrested by aphidicolin, a DNA polymerase inhibitor, than that of asynchronously growing HCT116 cells (Fig. 8a), whereas DNMT1 in G1 cells, released from mitotic shake-off after treatment of microtubule inhibitor nocodazole^55^, has a lower content of K142 methylation (Fig. 8b). Since G1 cells usually take about 9–10 h to progress from late mitotic phase to the end of G1 phase^26, 55^, we have tested the effects of LSD1 or PHF20L1 knockdown in early and mid-G1 phase cells after their release from mitotic shake-off. We found that DNMT1 appeared to be much resistant to the siRNA-mediated knockdown of LSD1 in G1 cells (0–8 h, Fig. 8c). In contrast, the protein level of DNMT1 in G1 cells was reduced if PHF20L1 was knockdown by specific siRNAs in the parallel experiments (Fig. 8d), even these cells contain relatively low levels of the methylated DNMT1 (Fig. 8b).

Our studies further revealed that S phase is a major cell cycle phase that the protein stability of DNMT1 is dynamically regulated. In cells synchronized at the G1/S border by sequential treatment with thymidine and aphidicolin^26^, we found that knockdown of LSD1 caused a dramatic downregulation of DNMT1 between 4–8 h, with a peak at about 6 h, after cells being released from the G1/S border (Fig. 8e), while DNMT1 appeared less sensitive to LSD1 deficiency in both early S phase (0–4 h) and late S or early G2 phase (8-10 h) cells. PHF20L1 also protected DNMT1 in S phase cells between 0 and 8 h after releasing from the G1/S border (Fig. 8f and Supplementary Figure 2D). The stability of DNMT1 protein became less sensitive to PHF20L1 knockdown between 8 and 10 h (Fig. 8f, Supplementary Figure 2D).

To analyze the regulation of DNMT1 protein by the L3MBTL3-CRL4^DCAF5^ system, we examined whether the methylated DNMT1 protein and its interaction with L3MBTL3 are regulated in S phase, using PHF20L1 as a comparison, in the cells synchronized at the G1/S border by thymidine and aphidicolin^26^. This is because both PHF20L1 and L3MBTL3 bind to the methylated K142 but with opposite consequences (Fig. 4c, g)^38^. Our results showed that while there were some DNMT1 methylations and the DNMT1-L3MBTL3 interactions in the arrested cells (0 h), the levels of methylated DNMT1 were relatively low in early S phase (0–5 h), increased and peaked at 8–9 h, and then started to decline 10–12 h post-release from the G1/S border (Fig. 8g). There was significant binding of PHF20L1 to DNMT1 in arrested cells (0 hour) and in cells between 0 and 9 h after the release. In contrast, there was minimum interaction between DNMT1 and L3MBTL3 in early S phase cells (0–5 h). Even when the levels of methylated DNMT1 increased at 8–9 h, L3MBTL3 binding to DNMT1 was barely detectable, although the interaction between PHF20L1 and DNMT1 remained significant. However, the binding of L3MBTL3 to DNMT1 became greatly increased to peak at 10–11 h in late S and early G2 phases, preceded with the disappearance of PHF20L1 from its association with DNMT1 at 10–12 h (Fig. 8g). Total levels of DNMT1 protein were slightly increased in most of S phase (0–10 h) but became greatly reduced between 11–12 h after its binding to L3MBTL3 reached the highest levels at 10–11 h. These studies indicate that the methylation-dependent proteolysis of DNMT1 is cell cycle regulated by PHF20L1 and L3MBTL3 in S phase and is consistent with our observation that loss of PHF20L1 reduced DNMT1 protein levels between 0 and 8 h after releasing form the G1/S border (Fig. 8f). These studies indicate that L3MBTL3 and PHF20L1 regulate the degradation of the methylated DNMT1 protein in a mutually exclusive manner.

### The methylated DNMT1 protein is a critical target of L3MBTL3 and DCAF5

Although most of our single siRNA transfection of L3MBTL3 and DCAF5 can achieve about 70–90% of target protein reduction (Figs. 3d, e and 4b, d), we also found that re-transfection of the same siRNA to the respective siRNA-treated cells can achieve more complete depletion of L3MBTL3 or DCAF5 expression in HCT116 cells (Supplementary Figure 4). Under these conditions, we repeatedly found that depletion of L3MBTL3 or DCAF5 alone is sufficient to induce the elevated level of DNMT1 protein and causes a reduction of proliferating cell numbers, as compared with that of a non-specific control siRNA against luciferase (Supplementary Figure 4). However, co-depletion of DNMT1, together with either L3MBTL3 or DCAF5 siRNAs, restored the cell numbers to that of control siRNA in L3MBTL3 or DCAF5 deficient cells (Supplementary Figure 4). Since knockdown of L3MBTL3 or DCAF5 induced elevated levels of methylated DNMT1 protein (Fig. 6a), these studies suggest that DNMT1 is a critical target of L3MBTL3 and the CRL4^DCAF5^ ubiquitin E3 ligase complex and that the elevated levels of methylated DNMT1 protein impede the growth of HCT116 cells.

### L3MBTL3 and CRL4^DCAF5^ also regulate the methylated E2F1 proteolysis

**Fig. 9:**
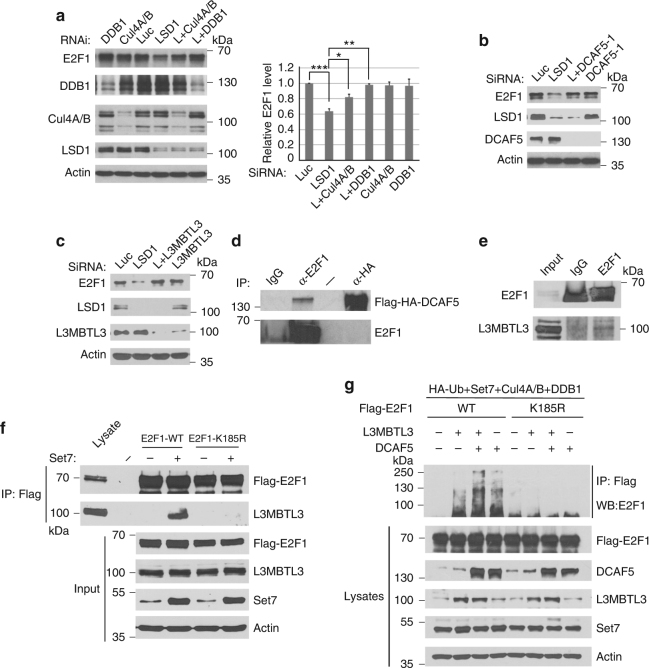
L3MBTL3 and CRL4^DCAF5^ regulate the methylation-dependent proteolysis of E2F1. **a** Downregulation of the CRL4 core complexes stabilized E2F1 in LSD1 knockdown cells. HCT116 cells were transfected with 50 nM siRNAs for each CRL4 core component, with or without LSD1 siRNA (LSD1 or L). E2F1 and indicated proteins were detected as indicated. Quantifications were represented by bar graph with mean and S.D. for error bars from three independent replicates, and Student’s t-test was calculated for significant differences, as described in Fig. 2a. *P < 0.05, **P < 0.01 and ***P < 0.001. **b** Downregulation of DCAF5 stabilizes E2F1. HCT116 cells were transfected with DCAF5 siRNA with or without LSD1 siRNA as indicated. E2F1, DCAF5, LSD1 and actin were examined by Western blotting, as in **a**. **c** Downregulation of L3MBTL3 prevents E2F1 proteolysis in LSD1 knockdown cells. HCT116 cells were transfected with control and L3MBTL3 siRNAs with or without LSD1 siRNA. E2F1, L3MBTL3, LSD1 and actin were examined by western blotting. **d** Ectopically expressed DCAF5 interacts with E2F1. Lysates from HCT116 cells expressing a stably expressed Flag-HA-DCAF5 were immunoprecipitated and blotted with indicated anti-HA and E2F1 antibodies. **e** L3MBTL3 and E2F1 interact with each other. Endogenous L3MBTL3 and E2F1 proteins were immunoprecipitated from HCT116 cells and western blotted with anti-E2F1 and L3MBTL3 antibodies as indicated. **f** The interaction between L3MBTL3 and E2F1 is dependent on the presence of K185 in E2F1. The Flag-wild-type E2F1 and Flag-K185R mutant expression constructs were transfected into HCT116 cells with or without SET7 expression as indicated. The interactions between L3MBTL3 and Flag-E2F1 or its K185R mutant were analyzed by anti-Flag and anti-L3MBTL3 immunoprecipitation and western blotting. Input: total cell lysates. **g** L3MBTL3 and CRL4^DCAF5^ ubiquitin E3 ligase complexes target E2F1 for polyubiquitination. Flag-E2F1 or Flag-K185R mutant expressing constructs were co-transfected into 293 cells together with vectors expressing HA-tagged ubiquitin (HA-Ub), SET7, CUL4A, CUL4B, and DDB1, in the presence or absence of L3MBTL3 and DCAF5 expressing constructs as indicated. The transfected proteins were immunoprecipitated with anti-Flag antibodies and Western blotted with anti-E2F1 antibodies. Experiments were repeated three independent times with the same conclusion

The consensus methylated K142 degradation motif in DNMT1 is present in many other non-histone proteins including the methylation motif containing lysine 185 (K185) in E2F1 (Fig. 1b)^8, 15^, a key transcription factor required for the expression of S phase genes in the cell cycle. SET7 is known to monomethylate K185 to trigger E2F1 proteolysis^15^, which is abolished by DNA damage or the presence of LSD1^15^. To characterize the regulation of methylated non-histone proteins more broadly, we tested whether L3MBTL3 and CRL4^DCAF5^ also regulate the methylation-dependent proteolysis of E2F1 (Fig. 9). Our studies revealed that while knockdown of LSD1 reduced E2F1 protein levels, co-silencing of either the components of CUL4^DCAF5^ or L3MBTL3 significantly stabilized E2F1 protein in LSD1 deficient cells (Fig. 9a–c, Supplementary Figure 2E, F). We also found that E2F1 binds to L3MBTL3 and DCAF5, and this binding was abolished when K185 is converted to Arginine (K185R), which cannot be methylated by SET7 (Fig. 9d, f). Expression of both L3MBTL3 and DCAF5 can also greatly stimulate the polyubiquitination of E2F1 protein, which was diminished in the K185R mutant (Fig. 9g). These studies indicate that the methylation-dependent polyubiquitination and proteolysis of E2F1 is also regulated by L3MBTL3 and CRL4^DCAF5^, similar to that of DNMT1.

### Mouse null deletion of *L3MBTL3* causes accumulation of DNMT1 protein

**Fig. 10:**
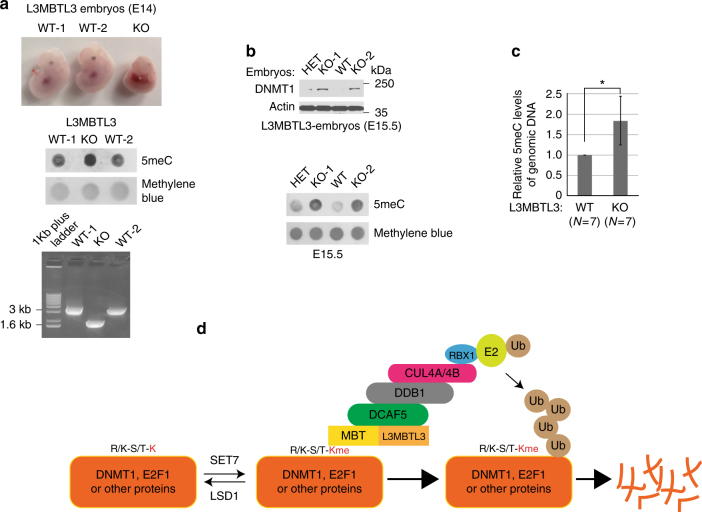
Effects of *L3MBTL3* null mutation in mouse embryos and a model for the degradation of methylated proteins. **a** Deletion of mouse *L3MBTL3* gene is embryonic lethal. The mouse wild-type (WT) and *L3MBTL3* null (−/−, KO) mutant embryos at embryonic day 14 (E14) after breeding. The genomic DNA from two wild-type and the KO embryos were extracted and used for genome-typing and 40 ng of the genomic DNA from each embryo was heat denatured, spotted onto the Genescreen membrane, and blotted with the anti-5-methylcytosine antibody (5mC) using the Methylene Blue staining as the loading control. **b** The levels of DNMT1 protein and methylated DNA increased in *L3MBTL3* KO mutants. The mouse wild-type (WT), heterozygous (HET), and *L3MBTL3* null (KO) mutant embryos at E15.5 from the same female mouse were used to analyze the levels of DNMT1 protein and methylated genomic DNA as indicated, with anti-DNMT1 and anti-5mC antibodies. **c** The relative methylated DNA content in *L3MBTL3* wild-type (+/+, *N* = 7) and KO (−/−, *N* = 7) mutant embryos from 6 different female mice between E14 and E15.5 was examined and compared. Statistically significant differences between means of methylated DNA levels in wild-type and knockout mutants were compared using two-tailed equal-variance independent Student’s *t*-test. *P < 0.05. The levels of methylated DNA in the wild-type embryos were normalized and set as 1.0. Quantifications were represented by bar graph with mean and standard deviation (S.D.) for error bars from 7 L3MBTL3 KO embryos (*N* = 7) containing the elevated methylated DNA, which also correlated with the increased levels of DNMT1 protein in these KO embryos (please see Supplementary Figure 5 for more examples). **d** A schematic model for the ubiquitin-dependent proteolysis of the methylated R/K-S/T-K motif-containing proteins by L3MBTL3 and CRL4^DCAF5^ ubiquitin E3 ligase complexes. The lysine residue (red and bold K) within the consensus R/K-S/T-K motif is dynamically methylated or demethylated by SET7 or LSD1, respectively. The monomethylated lysine residue (red and bold Kme) is recognized by the MBT domain of L3MBTL3. L3MBTL3 further interacts with the CRL4^DCAF5^ ubiquitin E3 ligase complex to target the methylated protein substrates for polyubiquitination-dependent proteolysis

Mouse null mutation of the *L3MBTL3* (*MBT-1*−/−) gene was reported to impair the maturation of hematopoietic system, reduce the expression of genomic imprinted CDK inhibitor *p57*^*Kip2*^ gene, and cause anemia and late embryonic lethality around E17.5-E19.5^47^. To determine whether the deletion of *L3MBTL3* in the mouse causes any effects on DNMT1, we have analyzed the knockout (KO or null) mutation of *L3MBTL3* (MBT-1, −/−) mice. Consistent with the original report^47^, we found that deletion of both alleles of the *L3MBTL3* (−/−) gene caused visible developmental defects of embryos around E14-15 and all of the KO mutant embryos died by E17.5–E19.5 (Fig. 10a). In *L3MBTL3* KO mutant embryos, increased levels of 5’-methylated cytosine in the genomic DNA and accumulation of DNMT1 protein were observed (Fig. 10a, Supplementary Figure 5A). Since each *L3MBTL3* (+/−) heterozygous breeding pair of female and male mice produces 6–8 embryos, usually only 1–2 *L3MBTL3* KO embryos and equal number of wild-type embryos, together with 3–4 heterozygous embryos, are produced by such a breeding pair. Our subsequent studies on the randomized *L3MBTL3* KO mutant embryos (*N* = 7) from 6 different female mice revealed that the protein levels of DNMT1 were elevated in all of 7 *L3MBTL3* KO embryos (Fig. 10b, c, Supplementary Figure 5), which correlated with the increased levels of methylated DNA in the same set of 7 *L3MBTL3* KO embryos between E14-E15.5 (Fig. 10b, c, Supplementary Figure 5), as compared with that of wild-type (total *N* = 7) or *L3MBTL3* heterozygous (−/ + ) embryos in the same litter. Sometimes, we also observed a slight increase in the methylated DNA level in the *L3MBTL3* heterozygous mutant embryos (Fig. 10b). Thus, our studies revealed that loss of *L3MBTL3* in the mouse caused the accumulation of DNMT1 protein and increased levels of methylated DNA. Our finding should help explain the defects of *L3MBTL3* KO mutation for altered expression of genomic imprinted genes such as the CDK inhibitor *p57*^*Kip2*^ gene in the fetal liver and the failure of hematopoietic development that causes anemia^47^, as DNMT1 and DNA methylation are known to play critical roles in regulating genome imprinting and hematopoietic stem cells and progenitor cells during hematopoietic development^56, 57^.

## Discussion

In this report, we found that LSD1 acts as a demethylase that removes the methyl group from the monomethylated K142 in DNMT1 to stabilize the protein (Fig. 1). Although the motif surrounding K142 is highly conserved between human and mouse DNMT1 proteins, a previous study reported that K1096 in the mouse DNMT1 protein serves as a substrate of SET7 in vitro^35^. However, mutation of K1096 in the mouse DNMT1 did not completely abolish methylation of mouse DNMT1 by SET7, suggesting additional SET7 methylation sites exist in DNMT1. In addition, the efficiency of LSD1 demethylation on the methylated K1096 was low, maximum at 30% of total K1096-methylated DNMT1. We found that unlike the K142A mutant, K1094R did not significantly stabilize human DNMT1 protein in LSD1 knockdown cells (Fig. 1). LSD1 can directly and efficiently demethylate the monomethylated K142 peptide in vitro (Fig. 1d). Our evidence strongly indicates that the methylated K142 of DNMT1 is a major substrate for LSD1 demethylase both in vitro and in vivo.

Increasing evidence indicates that many non-histone proteins contain the consensus methylation motif, R/K-S/T-K, that are recognized and methylated by SET7^8, 12, 13, 17^. A recent biochemical study using purified SET7 recombinant protein revealed that it can methylate a large number of novel non-histone proteins in vitro^11^. While these studies show that many non-histone proteins are methylated by lysine-specific methyltransferases, very little is known about how these methylation signals in non-histone proteins are recognized and processed. In this report, we have investigated the mechanism by which DNMT1 and E2F1, two best-characterized methylated non-histone proteins, are regulated by methylation-dependent proteolysis. We show that the methylation-dependent proteolysis of DNMT1 and E2F1 is regulated by a novel methyl-binding protein, L3MBTL3, and a previously uncharacterized ubiquitin E3 ligase, the CRL4^DCAF5^ complex. Our studies are consistent with a model, as schematically illustrated in Fig. 10d, that methylated proteins are recognized by the MBT domain of L3MBTL3, which recruits the CRL4^DCAF5^ ubiquitin E3 ligase complex, to target the methylated proteins for ubiquitin-dependent proteolysis.

Our studies further revealed that the methylation-dependent DNMT1 degradation is highly cell cycle regulated, likely associated with its heightened catalytic DNA methylation activities in S phase and additional epigenetic functions in other cell cycle phases. In S phase cells, both LSD1 and PHF20L1 protect the methylated DNMT1 protein by preventing the binding of L3MBTL3 to DNMT1. Upon the dissociation of PFH20L1 and possibly reduced LSD1 activities, L3MBTL3 binds to the methylated DNMT1 to target it for the L3MBTL3-dependent proteolysis in late S and G2 phases. The dual protection mechanisms of LSD1 and PHF20L1 ensure that the methylation and degradation of DNMT1 protein is tightly regulated by the L3MBTL3-CRL4^DCAF5^ system in the cell cycle. Notably, DNMT1 and E2F1 are also known to form a protein complex with HDAC1 and Rb^53^, and degradation of both proteins for gene expression by the L3MBTL3-CRL4^DCAF5^ complex may be warranted.

## Methods

### Cell culture

HCT116 cells and 293 cells were purchased from ATCC and cultured in RPMI-1640 or DMEM medium supplemented with 10% fetal bovine serum and 1% antibiotics (Invitrogen). The cells have been recently authenticated and tested negative for contamination based on the pseudo diploid genome for HCT116 cells and protein markers for both HCT116 and 293 cells. HCT116-Flag-DNMT1 knock-in cells were kindly provided by Dr. Zhenghe Wang (Case Western Reserve University) in which one of two *DNMT1* gene alleles was C-terminally tagged by the Flag epitope sequence^36^. All cells were negative for the mycoplasma tests. For stable expression, human DNMT1, L3MBTL3, and DCAF5 cDNAs were cloned into the retroviral pMSCV-3XFlag-3XHA vector (Clontech) to construct the amino-terminal tagged 3XFlag-3XHA fusion proteins. Transient transfection for recombinant viral packaging was conducted in 293 cells using the calcium phosphate method^30^. The recombinant viruses were collected from cell culture media of transfected cells and were used to infect HCT116 or 293 cells. Cells with stable expression of targeted proteins were selected by the puromycin resistance^32, 37^. Expression of target proteins was confirmed by western blotting analyses. Lipofectamine 2000 was used for other transient expression constructs into HCT116 or 293 cells such as the expression of the DNMT1 K142A mutant and in vivo polyubiquitination analyses^32^.

### Cell cycle synchronization

For synchronization of cells at the G1/S border, active growing HCT116 cells were treated with 2.5 mM thymidine for 18 h, released into fresh cell culture medium for 9 h, and then treated again with 5 μg/ml aphidicolin (Sigma) for another 15 h to synchronously arrest the cells at the G1/S border^26–28^. Cells were washed with the phosphate-buffered saline (PBS) and released into fresh culture medium for S phase entry. For synchronization in mitosis using the mitotic shake-off method, actively growing HCT116 cells were treated with 40 ng/ml nocodazole for 14 h and the round-up mitotic cells were shaken off the culture dishes, collected by centrifugation, and washed extensively with PBS to remove nocodazole^26, 55^. The cells were re-plated in fresh culture medium without the drug. The cells usually attached to the plates within 1–3 h after exit of mitosis to enter into the G1 phase, which is about 10–12 h.

### Antibodies and immunological analysis

Anti-LSD1 (A300-215A), L3MBTL3 (A302-852), DNMT1 (A300-041A), SET7 (A301-747A), and DDB1 (A300-462A) antibodies were purchased from Bethyl Laboratories; anti-PHF20L1 (HPA028417) were from Sigma; anti-E2F1 (KH95), cyclin E (HE12), cyclin B1 (GNS1), and actin (Sc-1616) antibodies were from Santa Cruz Biotechnologies. The anti-5-methycytosine monoclonal antibody (33D3, A-1014-050) was from Epigentek. Anti-CUL4A and CUL4B, Flag, HA, and GST antibodies were described before^28, 32, 37^. The monomethylated K142 (PLSKPRTPRRSKme1SDGEAKPC) and cognate unmethylated peptides of DNMT1, as well as a DCAF5 peptide (MKRRAGLGGSMRSVVGFLSQRGLHC) were synthesized at Selleck Chemicals. The monomethylated K142 peptide and DCAF5 peptide were used to raise rabbit polyclonal antibodies after coupling these peptides to keyhole limpet hemocyanin (KLH)^26–28^. For direct western blotting, the cells were washed with PBS and directly lysed in the 2XSDS sample buffer (4% SDS, 100 mM Tris, pH6.8, and 20% glycerol). Protein concentrations were measured by the protein assay dye (Bio-Rad) and equalized before addition of 0.2% bromophenol blue, freshly added 10% beta-mercaptoethanol, and boiled for 15 min. Usually three replicate repeats of siRNA-based knockdown experiments were conducted to evaluate the effects on target proteins. Each set of protein samples in a siRNA-based knockdown experiment were normalized by total proteins to the luciferase siRNA control and further analyzed by western blotting analysis in three replicates to normalize protein levels to the protein loading controls such as actin to evaluate knockdown efficiencies, effects on target proteins, and operational variables. The antibodies for western blotting were: anti-LSD1, CUL4A, CUL4B, HA, GST, Flag, and 5meC antibodies diluted at 1:2000; anti-L3MBTL3, DNMT1, SET7, DDB1, cyclin B1 and cylcin E antibodies at 1:1000; anti-PHF20L1 and E2F1 antibodies at 1:300, and anti-actin antibodies at 1:6000^26–28^. For immunoprecipitation, cells were lysed with the NP40-containing lysis buffer (0.5% NP40, 50 mM Tris, pH 7.5, 150 mM NaCl, and protease inhibitors, aprotinin, trypsin inhibitor, leupeptin, and benzamindine)^26–28^. Lysates were cleared by centrifugation and the proteins in the cell lysates were quantified by the protein assay dye (Bio-Rad). Usually 500 μg of lysates were used for each immunoprecipitation assay^48^. The antigen–antibody complexes were then pulled down by 30 μl Protein A-Sepharose (GE Healthcare) and specific proteins were detected by western blotting analyses^26–28^, using secondary goat anti-mouse HRP (Jackson Immuno Research, 115-035-008) and goat–anti-rabbit antibodies (Jackson Immuno Research, 111-035-008), or Protein A HRP (GE Healthcare, NA9120V), all at 1:2000 dilutions. The uncropped Western blot images were included in Supplementary Figure 6-19.

### Affinity purification of the monomethylated K142 peptide antibodies

The unmethylated and monomethylated K142 peptides were immobilized to Sulfolink-coupled-resins (Thermo Fisher) by covalently cross-linking with the cysteine residues at the end of the peptides to the resin. The anti-monomethylated K142 peptide serum (5 ml) was diluted in 1:1 in PBS and first passed through the unmethylated K142 peptide column (1 ml) to deplete anti-K142 peptide antibodies. The unbound flow-through antibody fraction was then loaded onto the monomethylated K142 peptide column (0.5 ml), washed, and the bound antibody fraction was eluted by 5 ml of 100 mM glycine, pH 2.5. The eluted antibodies (0.5 ml/fraction) were immediately neutralized by adding 100 μl of 2 M Tris, pH 8.5, and tested for specificity toward the monomethylated K142 peptide but not to the unmethylated K142 peptide^26–28^.

### RT–qPCR analysis

The mRNAs from cells were isolated by the TRIzol kit (Thermo Fisher Scientific), reverse-transcribed, and quantified by real time quantitative PCR using SYBR green on an ABI Prism 7300 System^32, 37^. The primers used for RT–qPCR: GAPDH forward and reverse: GGTAGGGAGTTCGAGACCAG and TCAACGCAGTTCAGTTAGGC; DNMT1 forward and reverse: GCGTTCCGGCTGAACAAC and GCATCTCCACGTCTCCCT. Mouse genotyping primers flanking the deleted exon of *L3MBTL3* (*MBT-1*) mice: TCAGTCTGGGCAGTGATGTC and TTGCACACTAAGGAAGGGAAC.

### Methylated peptide binding assays

The monomethylated K142 and cognate unmethylated peptides of DNMT1 were covalently coupled to the Sulfolink-coupled resins^27, 28, 48^ (Thermo Fisher) through the disulfide bond between the C-terminal cysteine of the peptides and the resins. The Glutathione-S-transferase (GST) was fused in frame to the amino-terminus of the full length L3MBTL3 or its D381N mutant in pGEXKG and the recombinant fusion proteins were expressed in *Escherichia coli* and purified by Glutathione Sepharose (GE Healthcare)^26, 27^. For peptide binding assays, 25–30 μl of peptide-coupled resin were prewashed with the binding buffer (0.1% NP40, 50 mM Tris-Cl, pH 7.5, 150 mM NaCl) and the resins were pre-blocked with 5 μg GST protein at room temperature for 2 h. The peptide-coupled resins were then incubated with 1.5 μg each of GST or indicative GST-fusion proteins in the binding buffer overnight at 4 °C. The beads were extensively washed (4–5 times) with PBS and the proteins associated with the resins were analyzed by western blotting with anti-GST antibodies^48^. In the case of cell lysates as the source of endogenous L3MBTL3 or DCAF5, HCT116 cells were lysed with the NP40-containing lysis buffer, cleared nuclear fractions, and 250–500 μg of soluble cell lysate proteins were used for each peptide resin binding assay^48^.

### Demethylation analysis

The glutathione-S-transferase (GST) and the GST-LSD1 (human) fusion protein were expressed in *E*. *coli* BL21 strain and purified by the Glutathione Sepharose resin. Purified 1 μg of control GST or GST-LSD1 proteins were incubated with 100 ng of the monomethylated K142 or unmethylated control cognate peptides for 2 h at room temperature and the resulting peptides were blotted onto nitrocellulose membrane. The demethylated peptides were detected by immuno-blotting with the affinity purified anti-monomethylated K142 antibody or the anti-DNMT1 antibody for total peptides.

### Immuno-affinity purification and mass spectrometry analysis

Cell lysates from twenty dishes (150 mm × 25 mm) of HCT116 cells containing the knock-in Flag-DNMT1 or equal dishes of control HCT116 cells were employed for immuno-affinity purification using 1 ml of the anti-Flag M2 affinity gel (Sigma, A2220)^28, 37^. The proteins in the Flag-DNMT1 immunocomplexes were separated on an SDS-PAGE gel. The protein bands were excised and trypsinized. Tryptic peptides derived from each gel slice were separated by an online C18 (New Objective, IntegraFrit^TM^ Column, ProteoPep^TM^C18) nano-flow reversed-phase liquid chromatography (Easy nano-liquid chromatography) connected to an LTQ Orbitrap XL mass spectrometer (Thermo Scientific) at 300 nl/minute with 75 min of linear gradients from 0 to 35% acetonitrile in 0.1% formic acid. The liquid chromatography eluent was directly nanosprayed into the LTQ Orbitrap XL mass spectrometer with an ionization voltage of 2.2 kV. During the chromatographic separation, the LTQ Orbitrap XL is operated in a data-dependent mode and under the direct control of Xcalibur (Thermo Scientific). The mass spectrometry (MS) data were acquired using the following parameters: 5 data-dependent collision-induced dissociation MS2 scan survey in the linear ion trap per every full scan in the Orbitrap with the resolution set to a value of 60,000; 35% normalized collision energy in CID;±2 Da isolation window. Proteomic data were analyzed by QualBrowser in Xcalibur and Proteome Discoverer^26–28^.

### Transfection and siRNAs

Oligofectamine (Life Technologies) was used for transfection of siRNAs into HCT116 or 293 cells for siRNA-mediated knockdown analysis^28, 32^. Typically, 50 nM of each siRNA or their combinations were transfected into target cells for 48 h and cells were directly lysed in SDS or NP40 lysis buffers. The knockdown effects were examined by western blotting with specific antibodies. For verification of the effects of new proteins, usually 2 or 3 independent siRNAs were designed to examine the knockdown efficiency and the consequences of knockdown on target proteins. The siRNAs for human genes are: CUL4A: CTGCAGAACTGATCGCAAA; CULB: AAGCCUAAAUUACCAGAAA; DDB1: AGGAAACTTTGAAGAGATT; LSD1: GGAAGAAGAUAGUGAAAAC; AKT1: GACGGGCACATTAAGATCA; PHF20L1: TGGGGTTGATGGTGCTGAA; DCAF5-1: GCUGCAGAAACCUCUACAA; DCAF5-2: ATCACCAACTTCTGACATA; L3MBTL3-1: GATGCAGATTCTCCTGATA; L3MBTL3-2: GGTACCAACTGCTCAAGAA; SET7: GGGCAGTATAAAGATAACA; and control siRNA for luciferase: CATTCTATCCTCTAGAGGA. All siRNAs were synthesized from GE Dharmacon.

### Animals

The *L3MBTL3* (*MBT-1*−/+, B6;129-L3mbtl3tm1Tmiy) mice was kindly provided by Dr. Miyazaki, Tokyo University. They were bred, housed, and handled in accordance with the animal protocols approved by the institutional IACUC committee at University of Nevada, Las Vegas. All animals were housed in filter-topped cages supplied with irradiated bedding in HEPA-filtered clean room. All procedures were conducted according to the National Institutes of Health (NIH) Guide for Care and Use of Laboratory Animals. Before any mouse experiments were performed, the experimental procedures were approved by the UNLV Institutional Animal Use and Care Committee (IACUC) and all experiments complied with all relevant ethical regulations. The UNLV IACUC is an AAALAC approved facility and meets the NIH Guide for the Care and Use of Animals. For embryo analyses, usually 3 pairs of the *L3MBTL3* (−/+) male and female mice (10–12 weeks old) in three cages, each with 1 male and 1 female, were bred in the late afternoon and the breeding plugs were examined in the female mice in next morning. The positive plugs were counted as embryonic day 1 (E1) and the pregnant female mice between E14 and E17.5 were killed by the primary method of CO_2_ asphyxiation, followed by cervical dislocation (secondary method), as approved by the institutional IACUC committee. The sample size was chosen on the basis of our experience on *L3MBTL3* mutant mice and on cultured cells in order to detect the DNMT1 protein for differences of at least 50% between the wild-type and mutant groups. Usually a single pregnant female mouse produced about 6–8 embryos, which segregated at the Mendelian ratio, usually with 1–2 *L3MBTL3* (−/−), 1–2 wild-type and 3–4 heterozygous *L3MBTL3* (−/+) embryos. In the experimental analyses for examination of protein and methylated DNA levels, the investigators were unaware of the genotypes of the experimental embryos. The investigators also randomly analyzed the wild-type, heterozygous and homozygous knockdown embryos. The *L3MBTL3* null embryos between E17.5 and 19.5 usually died and became disintegrated so they were excluded from protein analyses.

### Analysis of proteins and DNA from embryos

The experimental procedures for embryo isolation were approved by the UNLV Institutional Animal Use and Care Committee (IACUC). The embryos were dissected from the killed pregnant female mice, washed with PBS, and lysed in the NP40 lysis buffer^58^. The nuclear and cytosolic fractions were separated by centrifugation. Genomic DNA was isolated from nuclear pellets by Zymo genomic DNA-tissue prep kit and quantified. Proteins in the cytosolic suppernant of the lysates were quantified by protein assay dye (Bio-Rad), equalized, and boiled for 15 min after addition of 1% SDS and 5% beta-mercaptoethanol to the lysates. Proteins were resolved in protein gel and analyzed by western blotting. For detection of methylated DNA, genomic DNA was heat denatured, dot blotted to the Genescreen membrane (PerkinElmer Life Sciences), UV cross-linked, and analyzed by the anti-5-methycytosine monoclonal antibody.

### Statistical information

Experiments were usually performed with at least three independent repeats (biological replicates) to ensure the results. Statistical plot analyses were performed using Microsoft Excel. Protein bands were quantified using Image J. To quantify protein loading in each western blot analysis of a set of protein samples, the same protein samples were analyzed with three repeated loading experiments (technical replicates). For cell-based assays, triplicated repeats in the same set of cells (technical replicates) were measured and the experiments usually repeated in three independent experiments with independently cultured cells (biological replicates). Quantitative data are expressed by bar graph, with mean and standard deviation (S.D.) for error bars from independent replicates. For siRNA-mediated knockdown experiments, statistically significant differences or variations between means of double and single knockdowns were normalized to the luciferase siRNA control and compared using two-tailed paired Student’s t-test. For animal experiments, triplicated breeding was used to obtain statistically significant number of embryos; and statistically significant differences between means of protein or methylated DNA levels in the control wild-type and knockout mutants were compared using two-tailed equal-variance independent Student’s t-test. All other data were determined using a two-tailed equal-variance independent Student’s t-test. The data in all figures met the assumption of normal distribution for tests. Different data sets were considered to be statistically significant when the *P*-value was <0.05 (*), 0.01 (**) or 0.001 (***)^59^.

### Data availability

The authors declare that the data supporting the findings of this study are available within the paper and its supplementary information files. The data that support the findings of this study are also available from the corresponding author upon reasonable request.

## Electronic supplementary material

Supplementary Information
