# Supplementary material for: Methylated DNMT1 and E2F1 are targeted for proteolysis by L3MBTL3 and CRL4DCAF5 ubiquitin ligase
Source: Nat Commun. 2018 Apr 24;9:1641. doi: 10.1038/s41467-018-04019-9 (PMC5915600; doi:10.1038/s41467-018-04019-9)
Supplement: Supplementary file 1 — Supplementary Information [file 41467_2018_4019_MOESM1_ESM.pdf]

Supplementary Information

Methylated DNMT1 and E2F are Targeted for Proteolysis by L3MBTL3 and CLR4^{DCAF5} Ubiquitin Ligase

Leng et al.

*Corresponding Author. Email: hui.zhang@unlv.edu.

Supplementary Figure 1. Characterization of the methylation of K142 in DNMT1 and the interaction between L3MBTL3 and CRL4 complexes.

Supplementary Figure 2. Regulation of DNMT1 by L3MBTL3 and L3MBL1 and L3MBTL2 do not bind to methylated K142 resins.

Supplementary Figure 3. Mapping the domains in L3MBTL3 and DCAF5 for their interaction.

Supplementary Figure 4. DNMT1 is a critical cellular target of L3MBTL3 or DCAF5.

Supplementary Figure 5. Increased levels of DNMT1 protein and methylated genomic DNA in the L3MBTL3 knockout mouse embryos.

Supplementary Figure 6-19. Uncropped Images of Western blots from Figures.

Supplementary Table 1: Peptides identified by Mass Spectrometry.

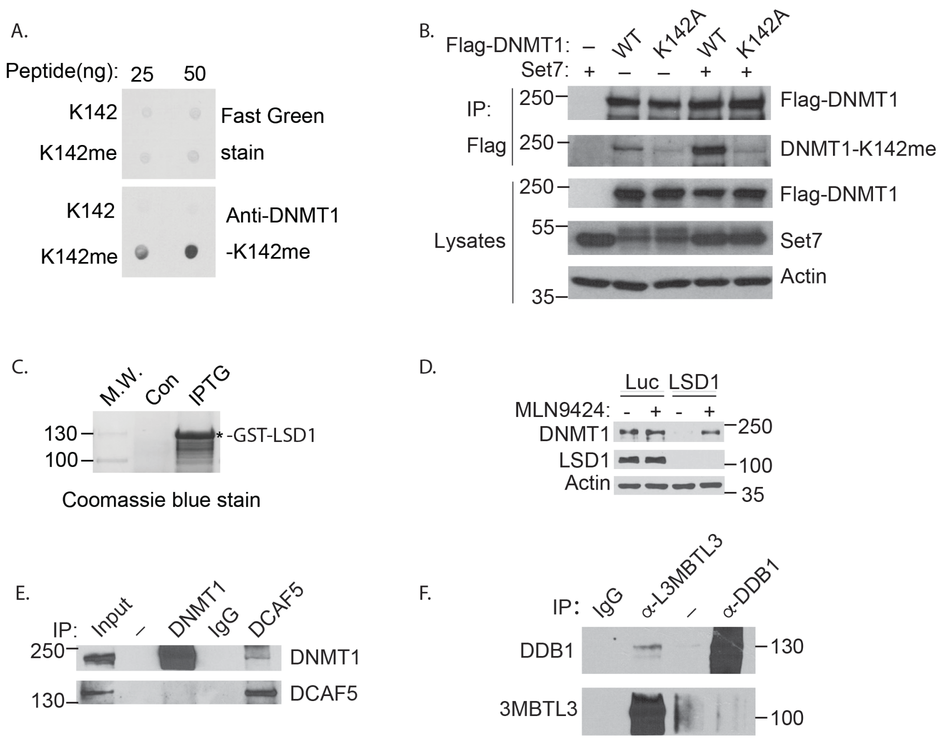

Supplementary Figure 1. Characterization of the methylation of K142 in DNMT1 and the interaction between L3MBTL3 and CRL4 complexes. A. Detection of the methylated K142 in DNMT1 by anti-methylated K142 antibodies *in vitro*. DNMT1 peptides containing the methylated K142 (K142me) or the cognate unmethylated K142 were spotted onto nitrocellulose membrane and Western blotted with anti-methylated K142 antibodies. Fast green stains for total peptides. B. The methylated K142 of DNMT1 is recognized by anti-methylated K142 antibodies *in vivo.* The Flag-DNMT1 and K142A mutant, with or without SET7, were expressed in HCT116 cells, immunoprecipitated with anti-Flag antibodies, and Western blotted with indicated antibodies. C. The GST-LSD1 protein expressed in BL21 strain of *E. coli*. The control is bacterial lysates without GST-LSD1. D. HCT116 cells were treated with 1 μM MLN9424 for 5 hours and the protein levels of DNMT1, LSD1, and actin were analyzed. E. DCAT5 interacts with DNMT1. The endogenous DCAF5 and DNMT1 proteins were immunoprecipitated from HCT116 cells and Western blotted with by anti-DCAF5 and DNMT1 antibodies. F. L3MBTL3 interacts with DDB1. The endogenous L3MBTL3 and DDB1 proteins were immunoprecipitated from HCT116 cells by anti-L3MBTL3 and DDB1 antibodies. Their interaction was examined by Western blotting. All experiments were repeated three independent times with the same conclusion and one example is shown.

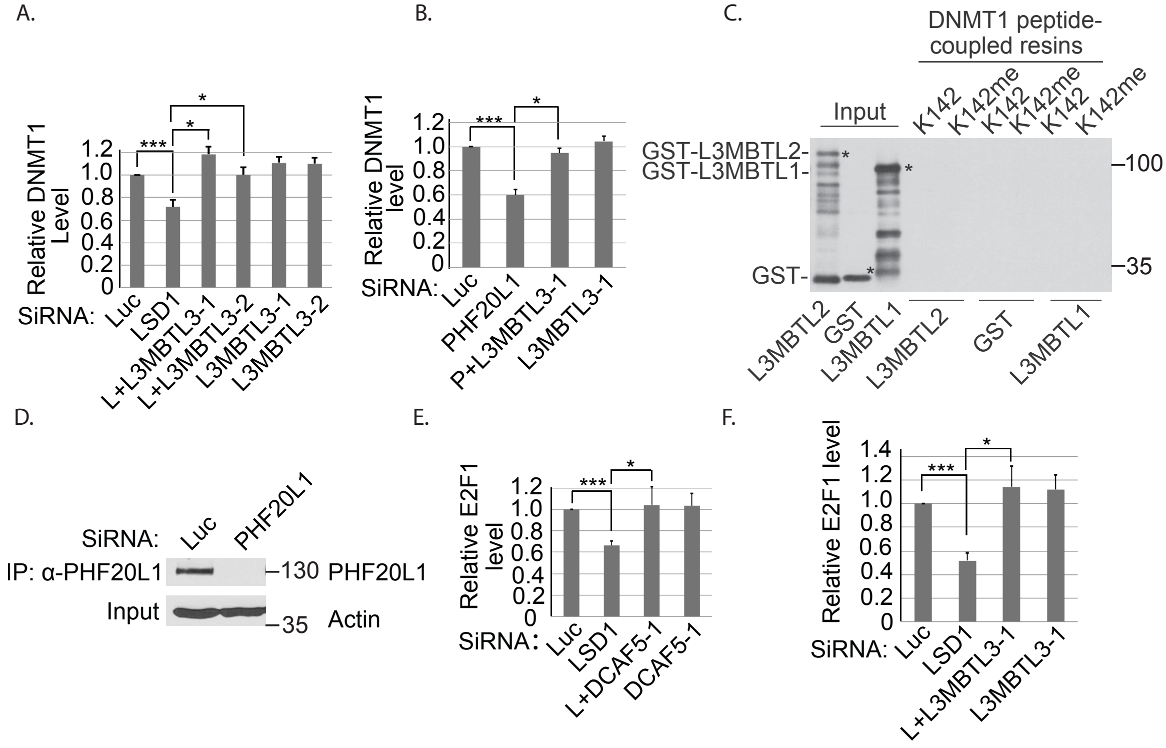

Supplementary Figure 2. Regulation of DNMT1 by L3MBTL3 and L3MBL1 and L3MBTL2 do not bind to methylated K142 resins. A. Quantification of protein blots in Fig. 4B. The protein band intensities were quantified as described in Fig. 2A. The P values of double to single knockdowns were calculated by paired student's t-test. *P<0.05 and ***P<0.001. B. Quantification of protein blots in Fig. 4C. The P values of double to single knockdowns were calculated by paired student's t-test. *P<0.05 and ***P<0.001. C. GST-The L3MBTL1 and GST-L3MBTL2 do not bind to the monomethylated K142 (K142me) peptide. The recombinant GST, GST-L3MBTL1, and GST-L3MBTL2 proteins expressed in bacteria were affinity purified by glutathione resins. Equal amounts of GST, GST-L3MBTL1 or GST-L3MBTL2 proteins (1.5 μg) were incubated with the peptide resins containing either the unmethylated K142 (K142me0) or monomethylated K142 (K142me) and examined for their specific interaction with the peptides by blotting with an anti-GST antibody, as described in Fig. 5D. The full length of GST or GST-fusion proteins is indicated by *. D. The knockdown efficiency of PHF20L1 siRNA in Fig. 8F. The PHF20L1 protein was immunoprecipitated from control and PHF20L1 siRNA treated cells and Western blotted with the anti-PHF20L1 antibody. E. Quantification of protein blots in Fig. 9B as in Fig. 2A. The P value was calculated by independent t-test and the P values of double to single knockdowns were calculated by paired student's t-test. *P<0.05 and ***P<0.001. F. Quantification of protein blots in Fig. 9C, conducted as in Figure 2A. *P<0.05 and ***P<0.001.

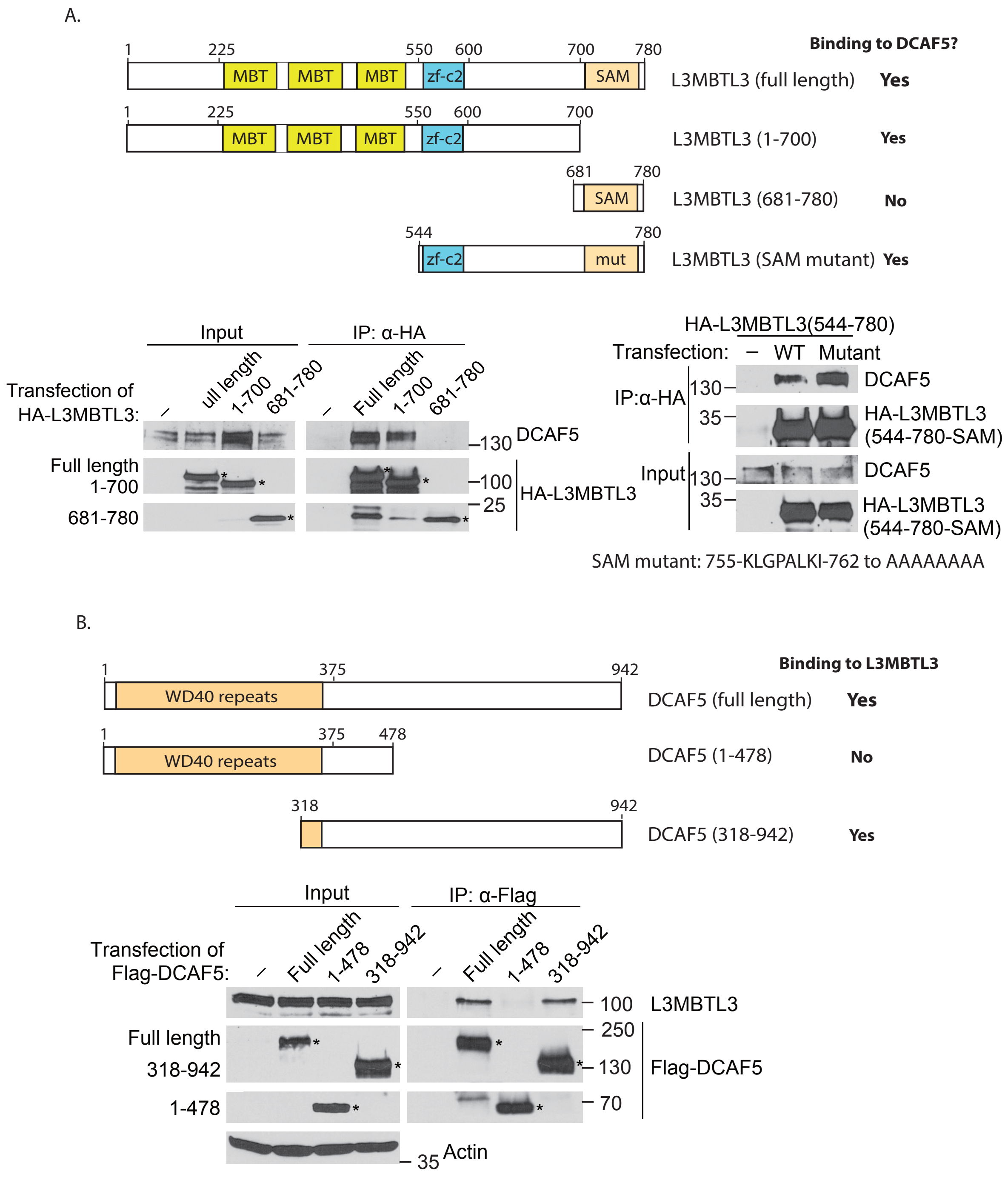

Supplementary Figure 3. Mapping the domains in L3MBTL3 and DCAF5 for their interaction. A. Top panels: schematic illustration of L3MBTL3 and mutants. Bottom panels: HCT116 cells were transfected with HA-tagged L3MBTL3 or various L3MBTL3 mutants and their interaction with Flag-DCAF5 were analyzed, as in Fig. 7. B. Top panels: schematic illustration of DCAF5 and mutants. HCT116 cells were transfected with the Flag-tagged DCAF5 or its mutants and their interaction with HA-tagged L3MBTL3 were analyzed.

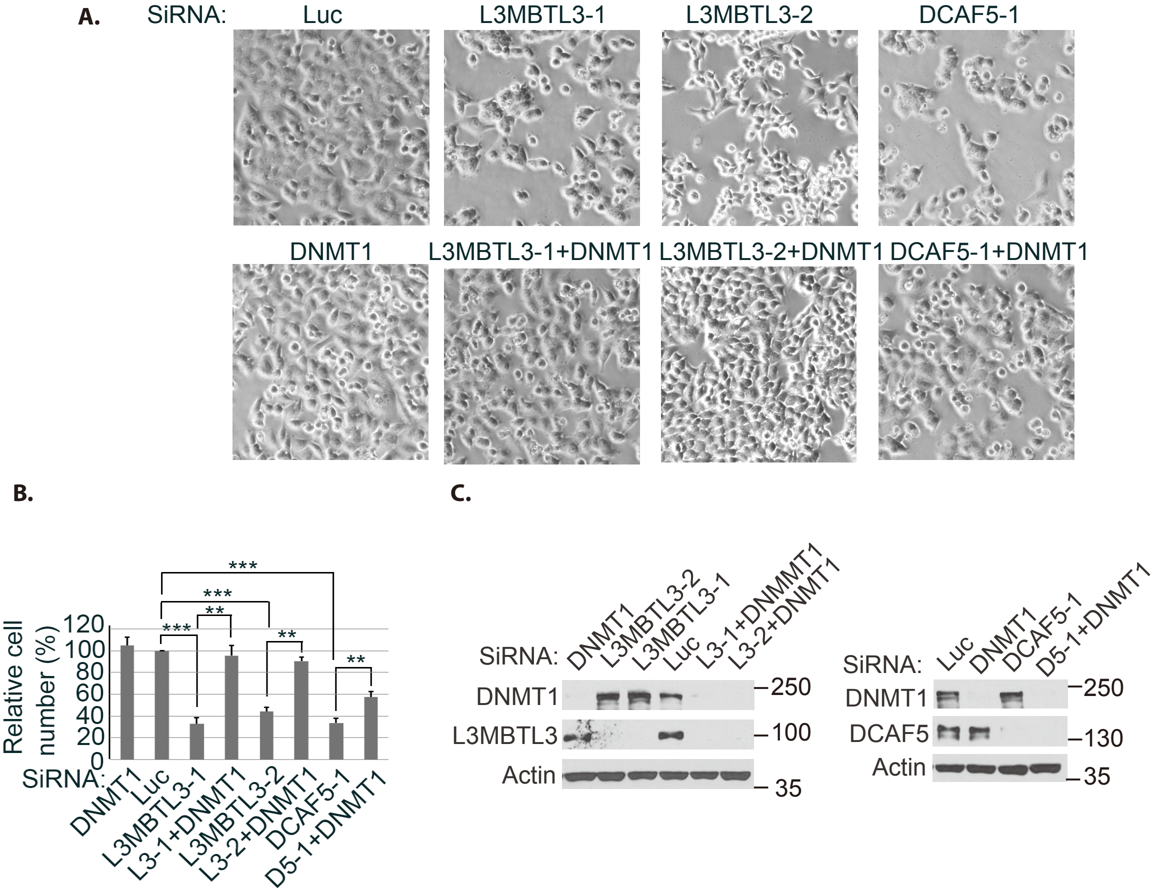

Supplementary Figure 4. DNMT1 is a critical cellular target of L3MBTL3 or DCAF5.

A. HCT116 cells were transfected 50 nM of control (luciferase, Luc) siRNA, two independent L3MBTL3 siRNAs, and a DCAF5 siRNA, with or without a DNMT1 siRNA for 48 hours as indicated. To effectively remove the endogenous protein expression, the siRNA-treated cells were transfected again with the same combination of siRNAs for additional 24 hours. **B.** Triplicated transfection cells from A were trypsinized, diluted, and blindly spotted onto a hemacytometer. Cells in four corners of hemacytometer were counted and to obtain average cells per dish. The differences between single (L3MBTL3, DCAF5, and DNMT1) and double knockdown (L3MBTL3+DNMT1 and DCAF5+DNMT1) cells, as compared with control siRNA treated cells, were plotted. Statistically significant differences between means of double and single knockdowns, normalized to control siRNA, in replicates were compared using two-tailed paired t-test and were statistically significant when the P-value was 0.01 (**) or 0.001 (***) (**$P<0.01$ and ***$P<0.001$). **C.** Western blotting was used to examine the effects of siRNA-based knockdown of each L3MBTL3, DCAF5, and DNMT1 protein from cells in A.

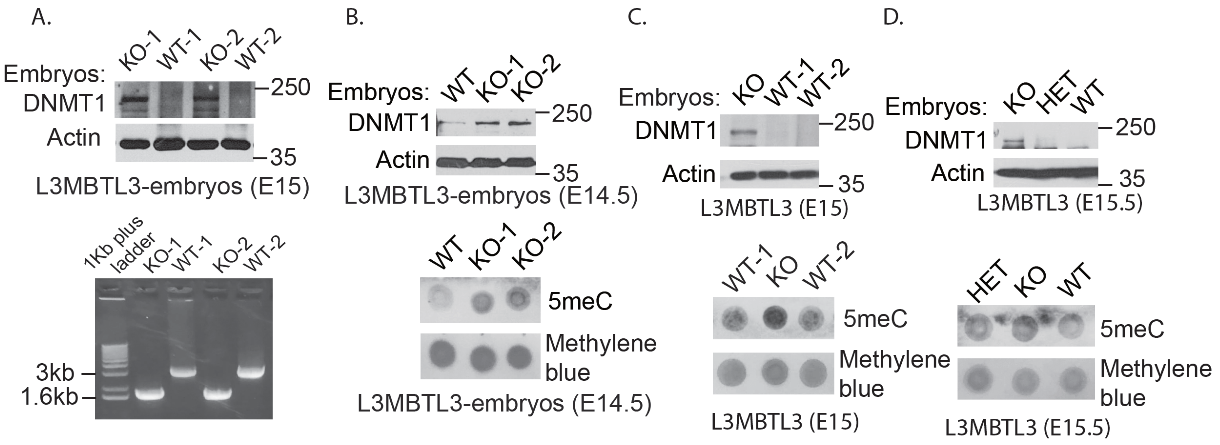

Supplementary Figure 5. Increased levels of DNMT1 protein and methylated genomic DNA in the L3MBTL3 knockout mouse embryos. A-D. Examples of independent batches of mouse wild-type (WT), heterozygous (HET), and *L3MBTL3* null (KO) mutant embryos between E14.5-E15.5, obtained from 6 heterozygous L3MBTL3 (+/-) pregnant female mice bred with heterozygous L3MBTL3 (+/-) male mice, as described in Fig. 10C, were used to analyze the levels of DNMT1 protein and methylated genomic DNA, as indicated, with anti-DNMT1 and anti-5meC antibodies. Total 42 embryos were produced by 6 different female mice, including 9 wildtype, 24 heterozygous embryos, 7 L3MBTL3 KO embryos that showed accumulated DNMT1 and increased methylated DNA, one of L3MBTL3 KO embryos only with elevated DNMT1, and one of L3MBTL3 KO embryo only with increased levels of methylated DNA (total 9 KO embryos). Please see Fig. 10A-C for more information on wildtype (N=7) and L3MBTL3 KO mutant embryos (N=7), which were part of this group of 42 total embryos from 6 L3MBTL3 (+/-) pregnant female mice.

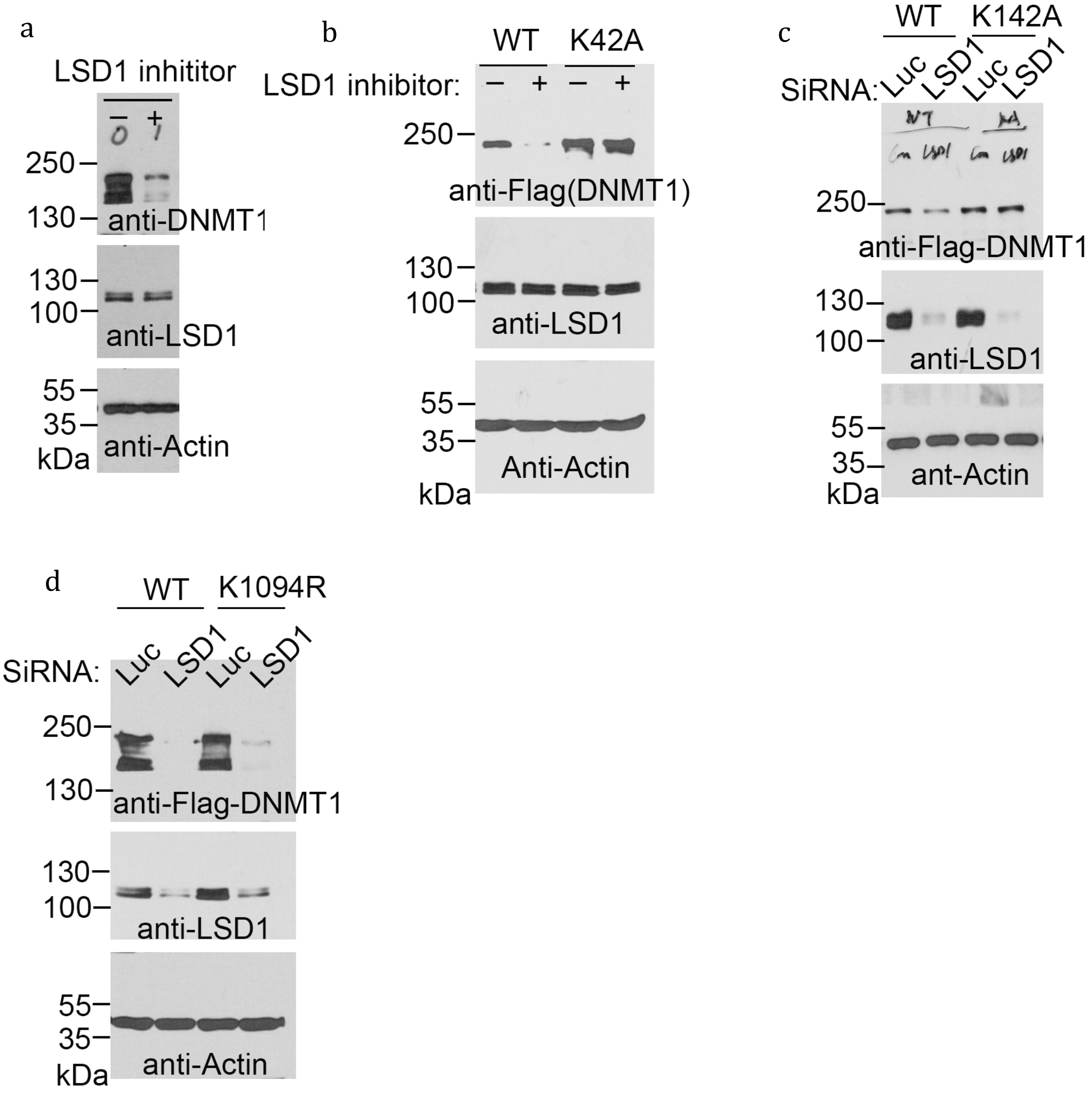

Supplementary Figure 6. Uncropped Images of Western blots from Figure 1
Uncropped Western blots from Fig. 1A(a), Fig. 1C(b), Fig. 1E(c), Fig. 1F(d).

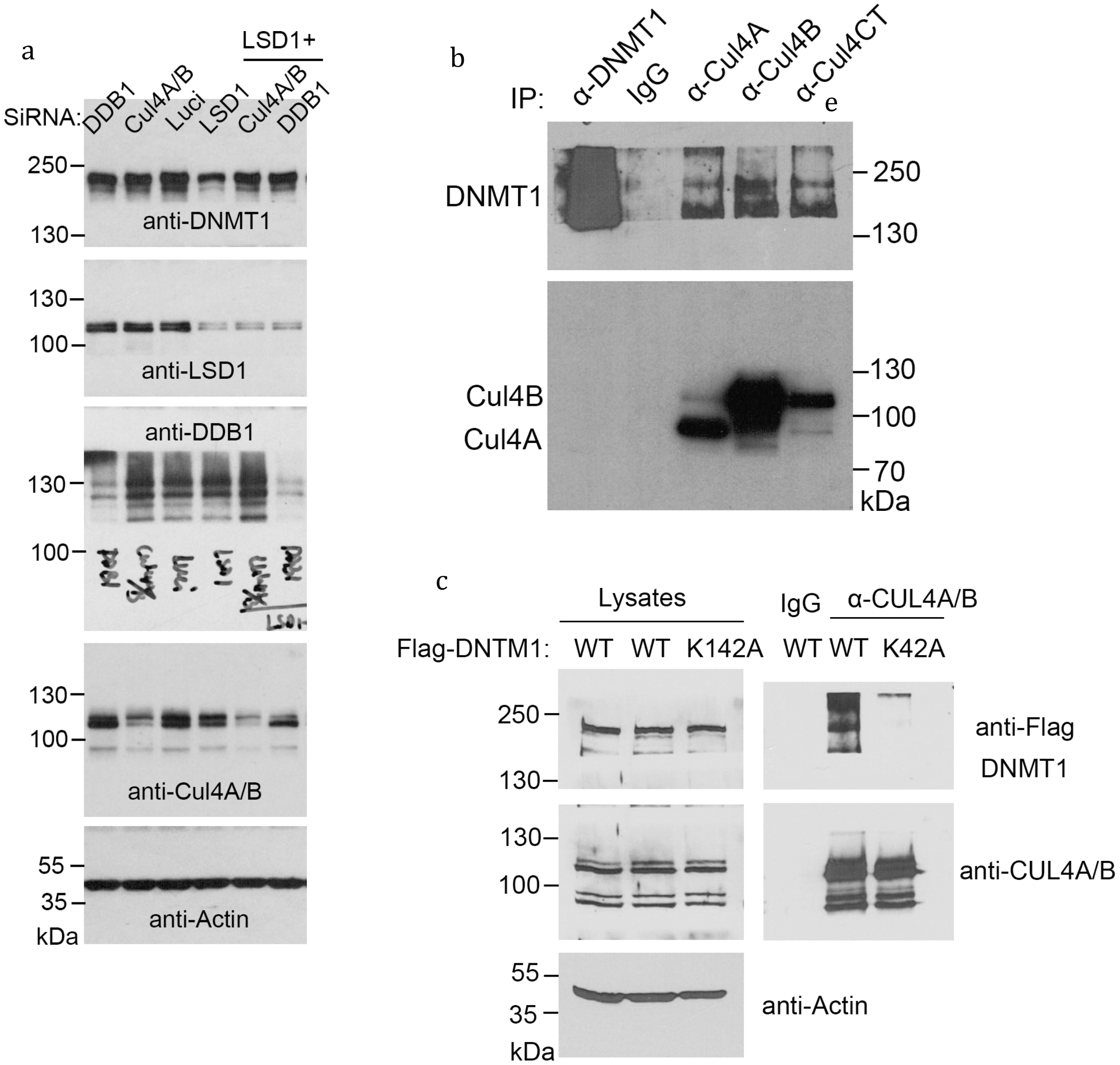

Supplementary Figure 7. Uncropped Images of Western blots from Figure 2
Uncropped Western blots from Fig. 2A(a), Fig. 2C(b), Fig. 2D.

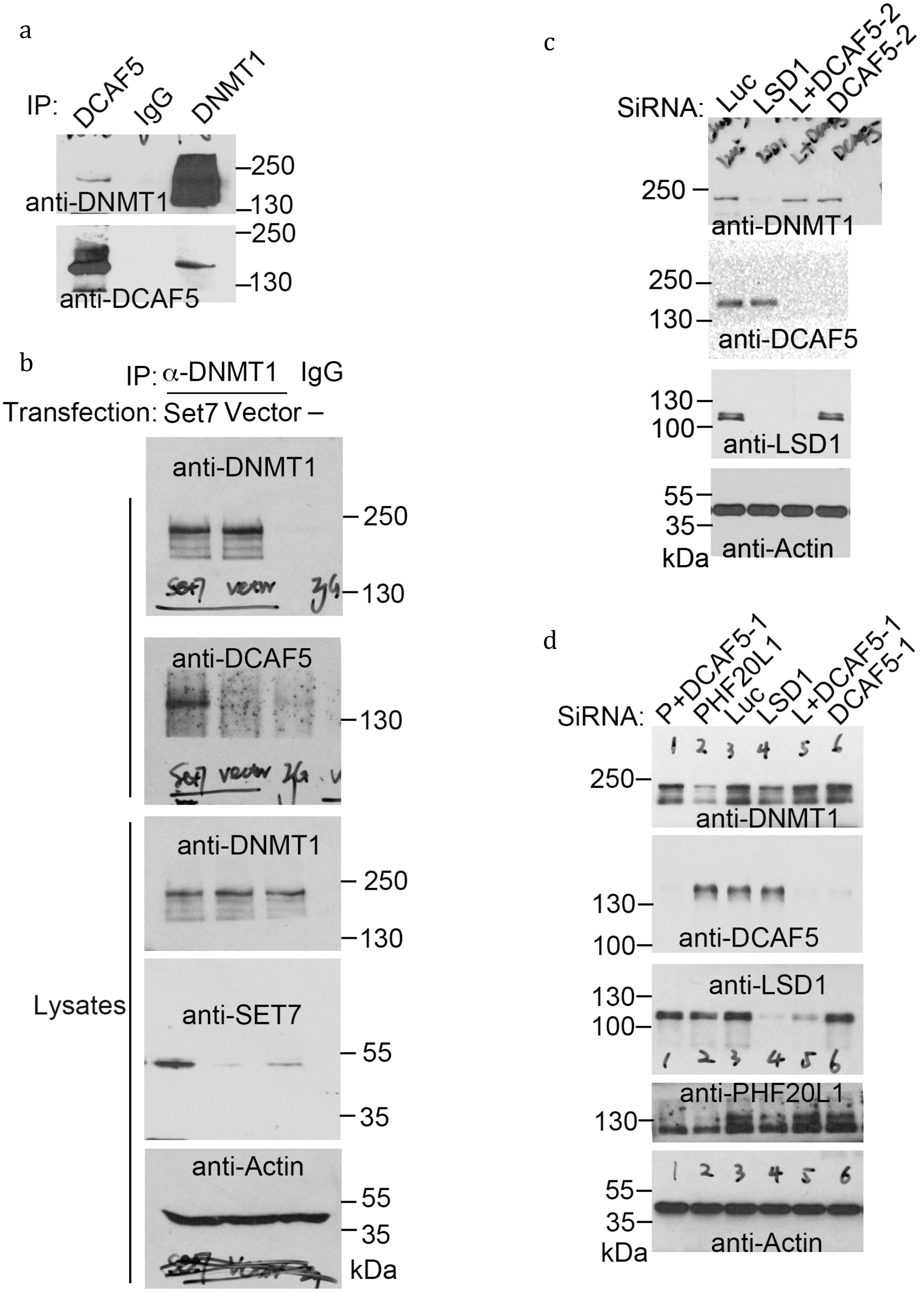

Supplementary Figure 8. Uncropped Images of Western blots from Figure 3
Uncropped Western blots from Fig. 3B(a), Fig. 3C(b), Fig. 3D(c), 3E (d).

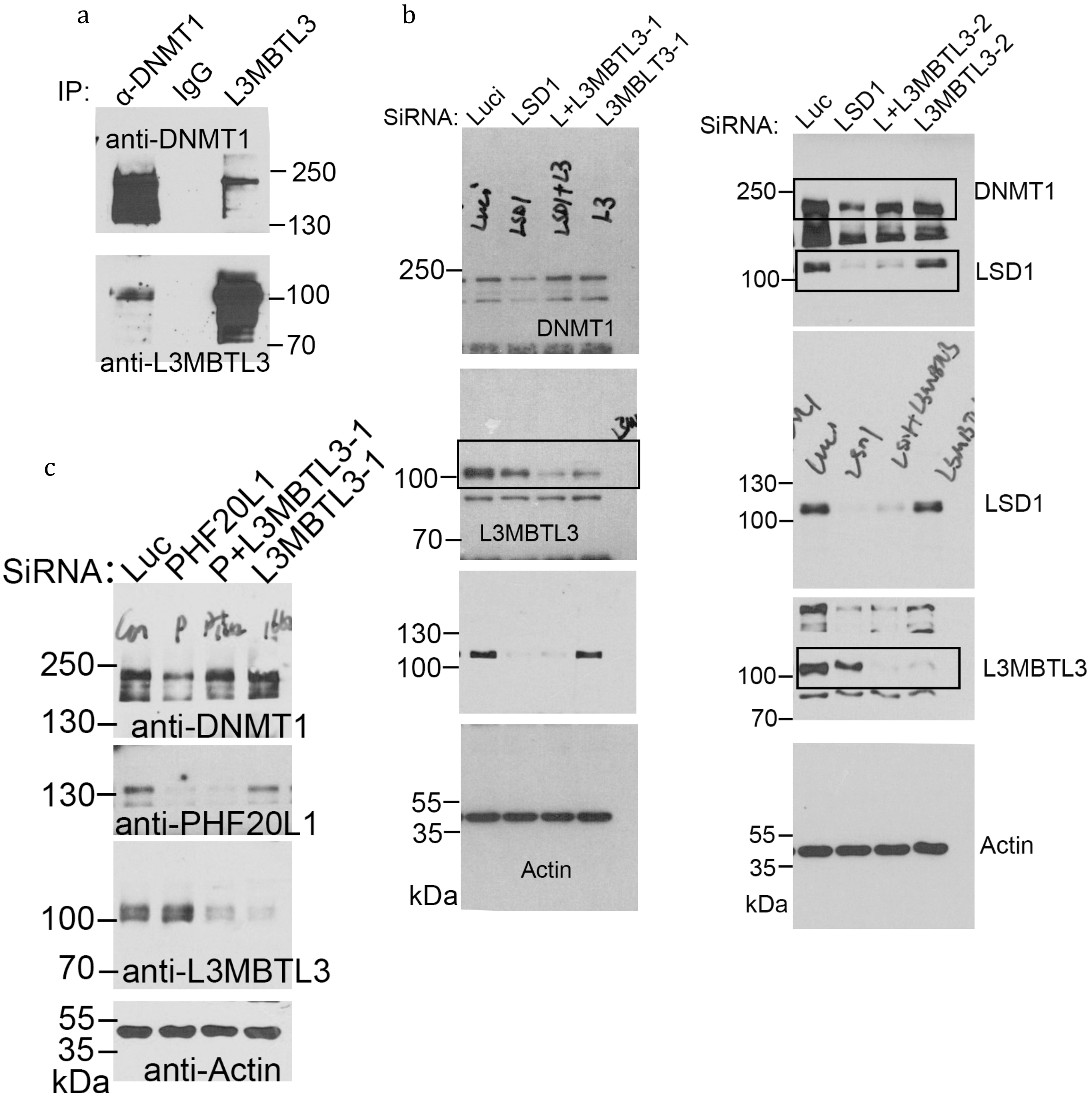

Supplementary Figure 9. Uncropped Images of Western blots from Figure 4(A-C)
Uncropped Western blots from Fig. 4A(a), Fig. 4B(b), Fig. 4C(c).

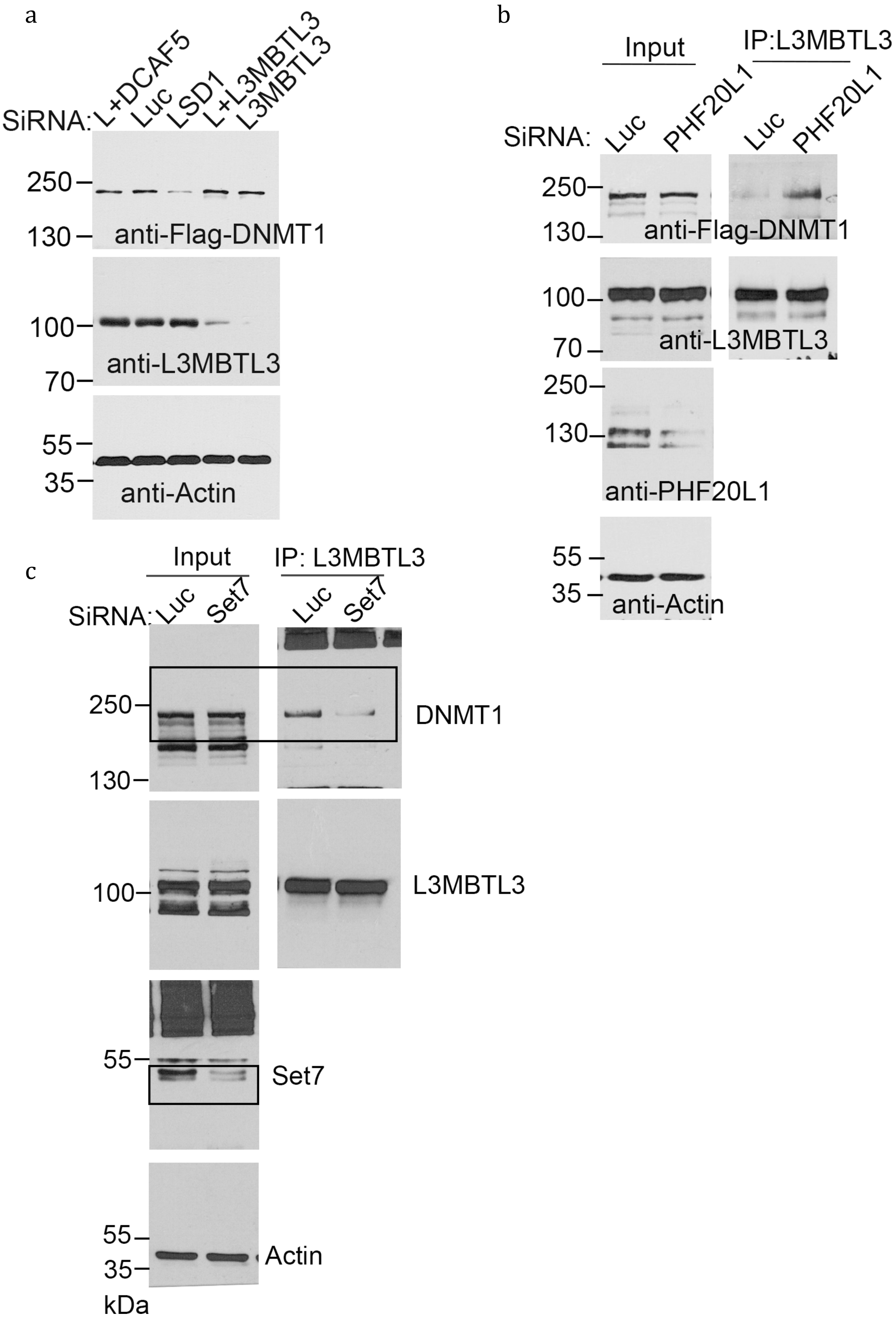

Supplementary Figure 10. Uncropped Images of Western blots from Figure 4D-G
Uncropped Western blots from Fig. 4D(a), Fig. 4F(b), Fig. 4G(c).

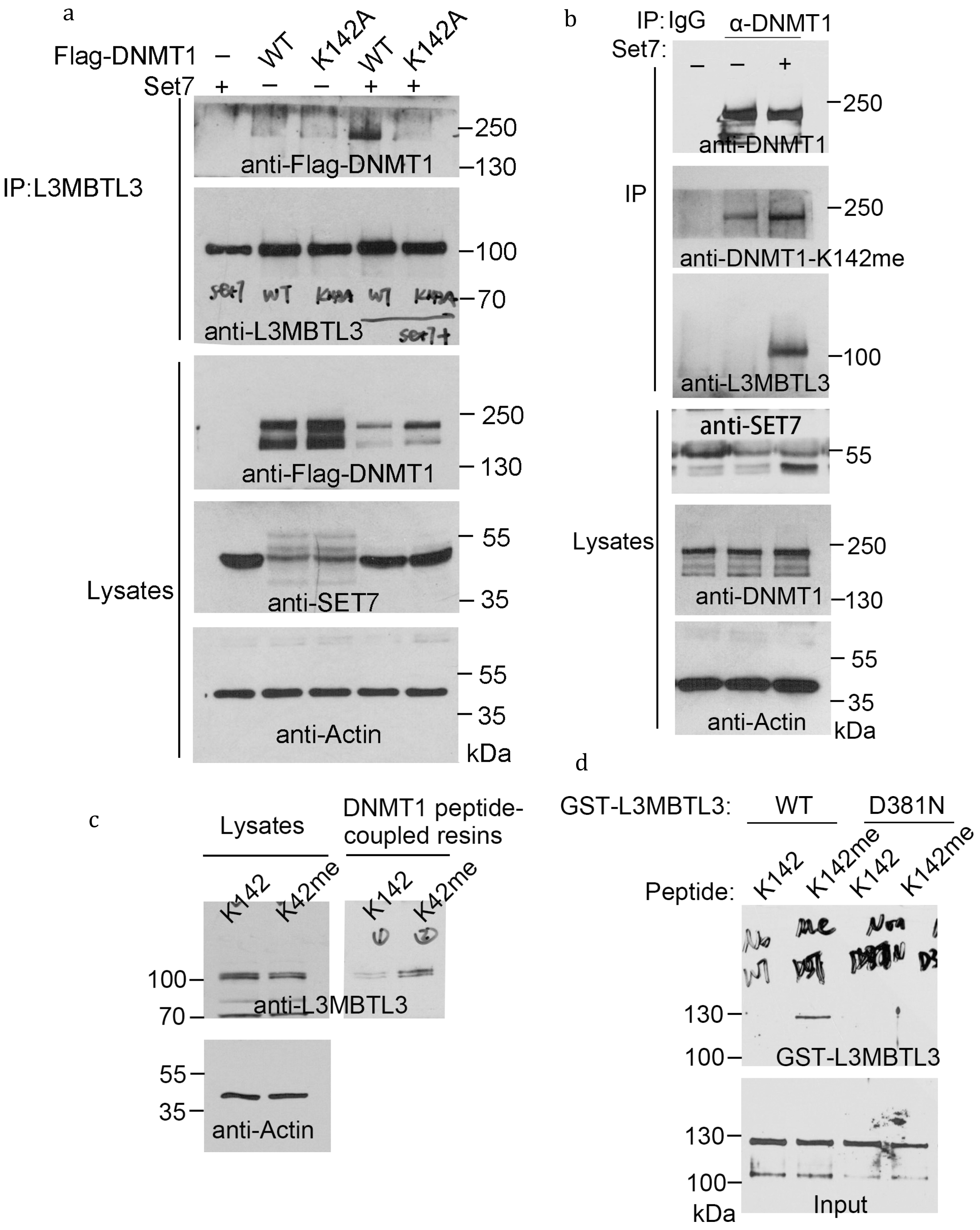

Supplementary Figure 11. Uncropped Images of Western blots from Figure 5A-D
Uncropped Western blots from Fig. 5A(a), Fig. 5B(b), Fig. 5C(c), 5D(d).

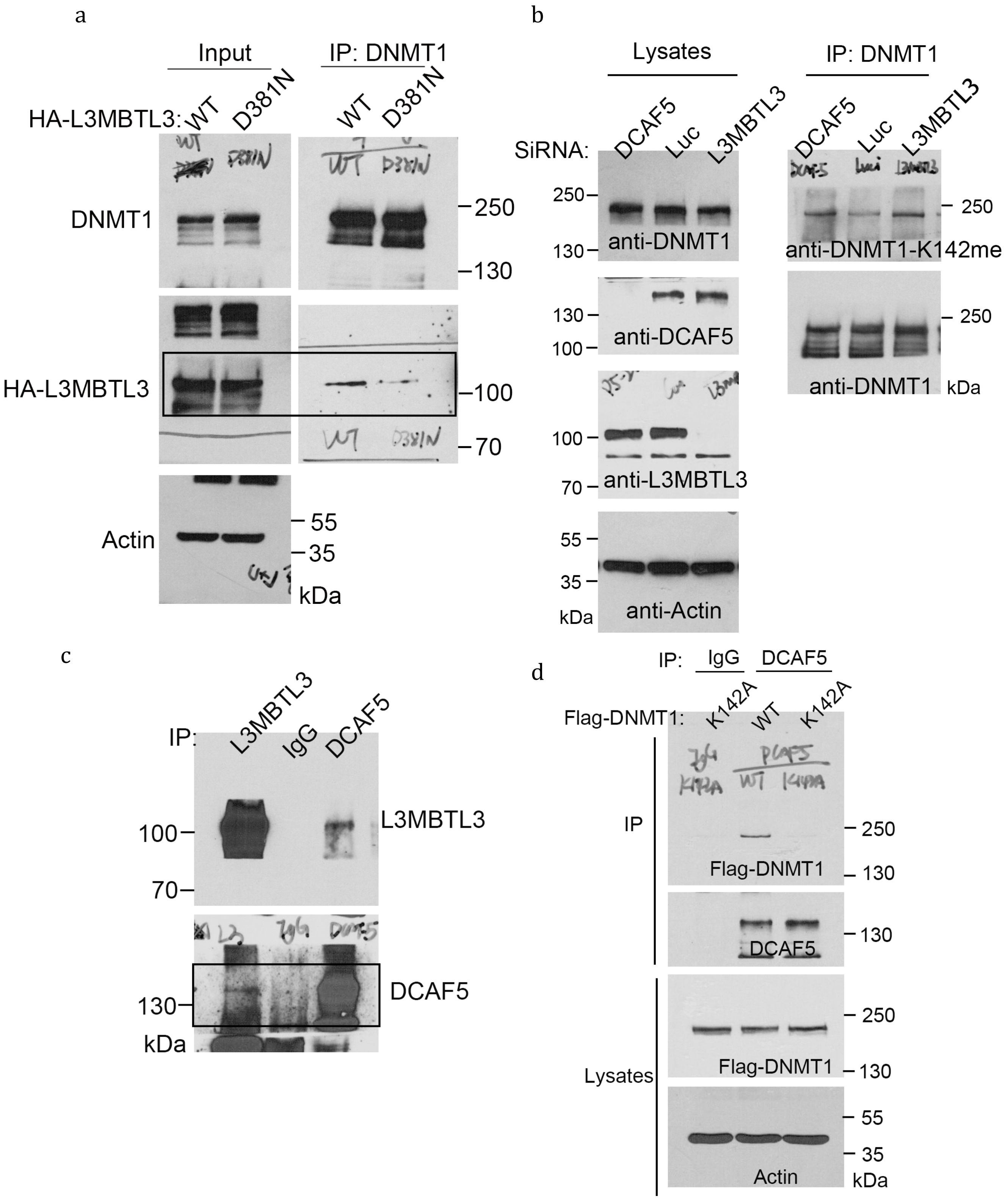

Supplementary Figure 12. Uncropped Images of Western blots from Figure 5E and Figure 6A-C. Uncropped Western blots from Fig. 5E(a), Fig. 6A(b), Fig. 6B(c), Fig. 6C(d).

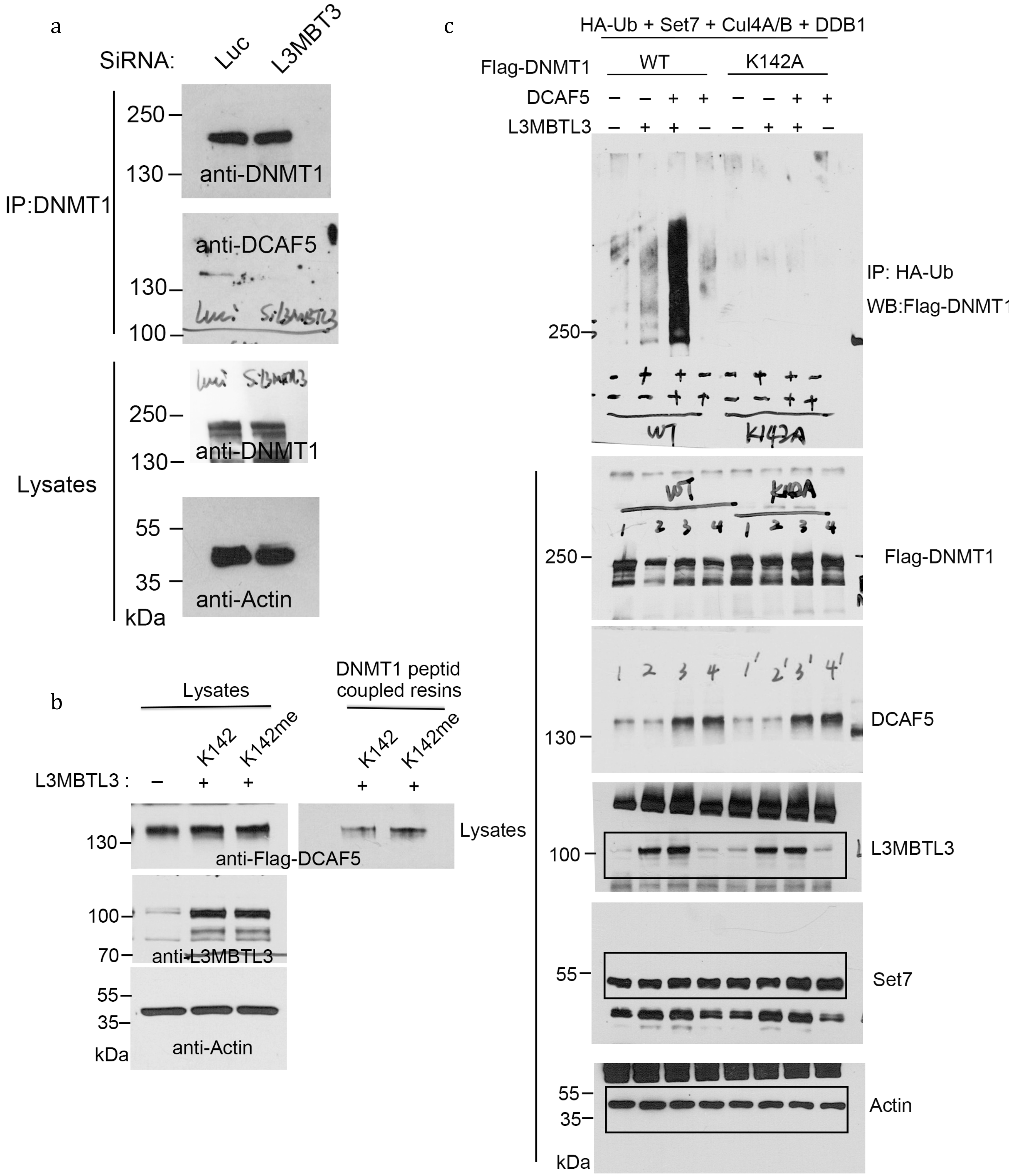

Supplementary Figure 13. Uncropped Images of Western blots from Figure 6D-F
Uncropped Western blots from Fig. 6D(a), Fig. 6E(b), Fig. 6F(c).

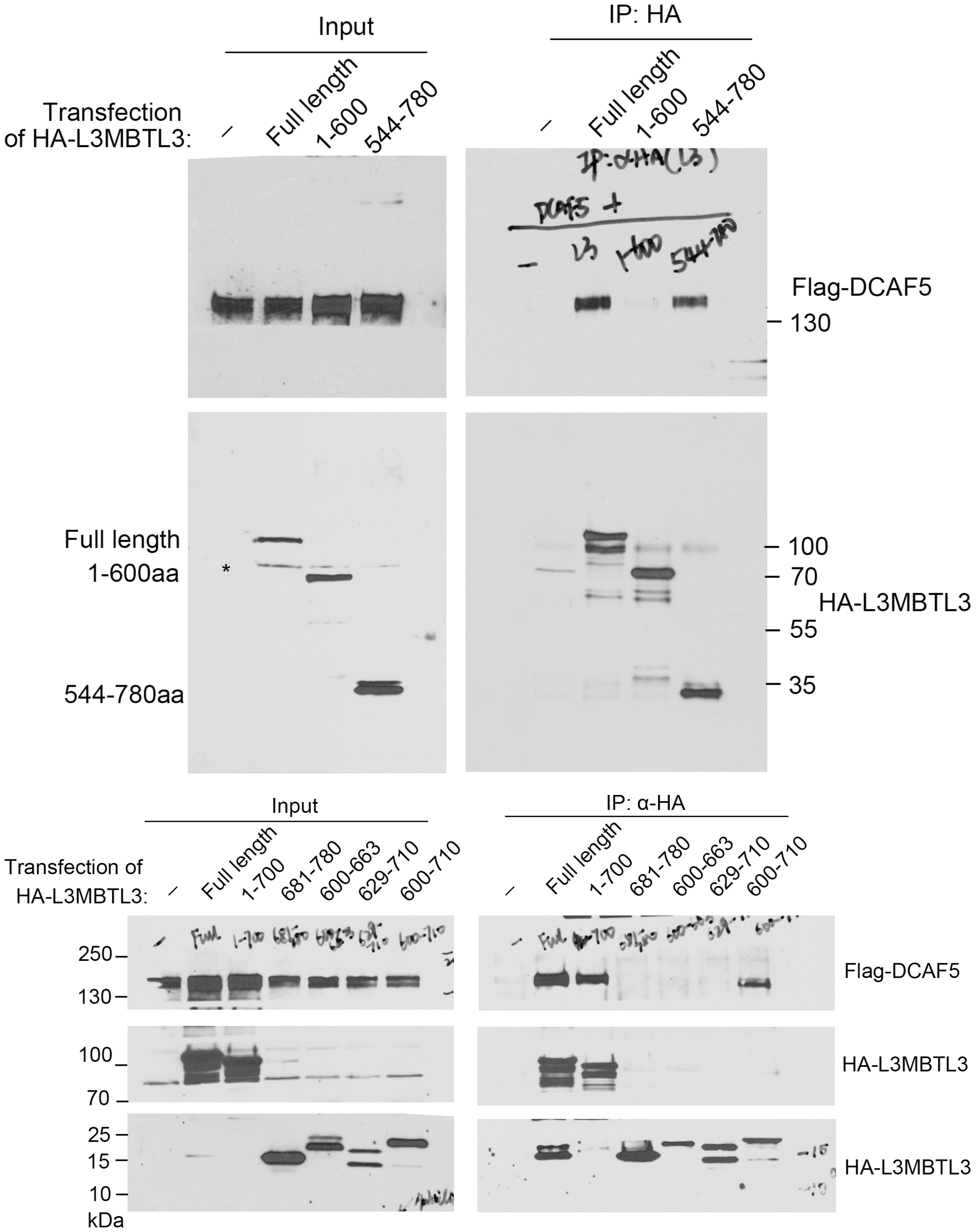

Supplementary Figure 14. Uncropped Images of Western blots from Figure 7B

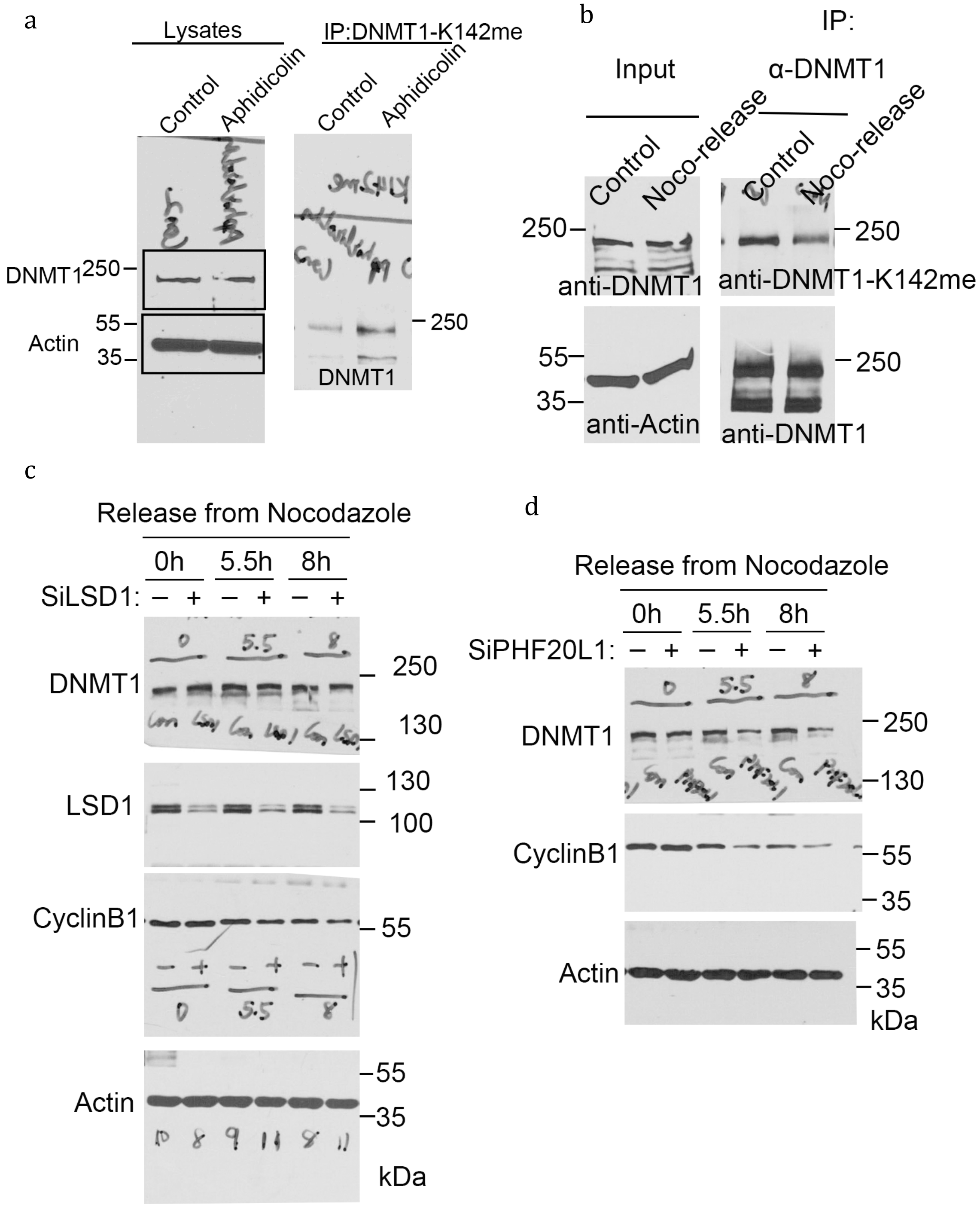

Supplementary Figure 15. Uncropped Images of Western blots from Figure 8A-D
Uncropped Western blots from Fig. 8A(a), Fig. 8B(b), Fig.8C(c), Fig. 8D(d).

a b

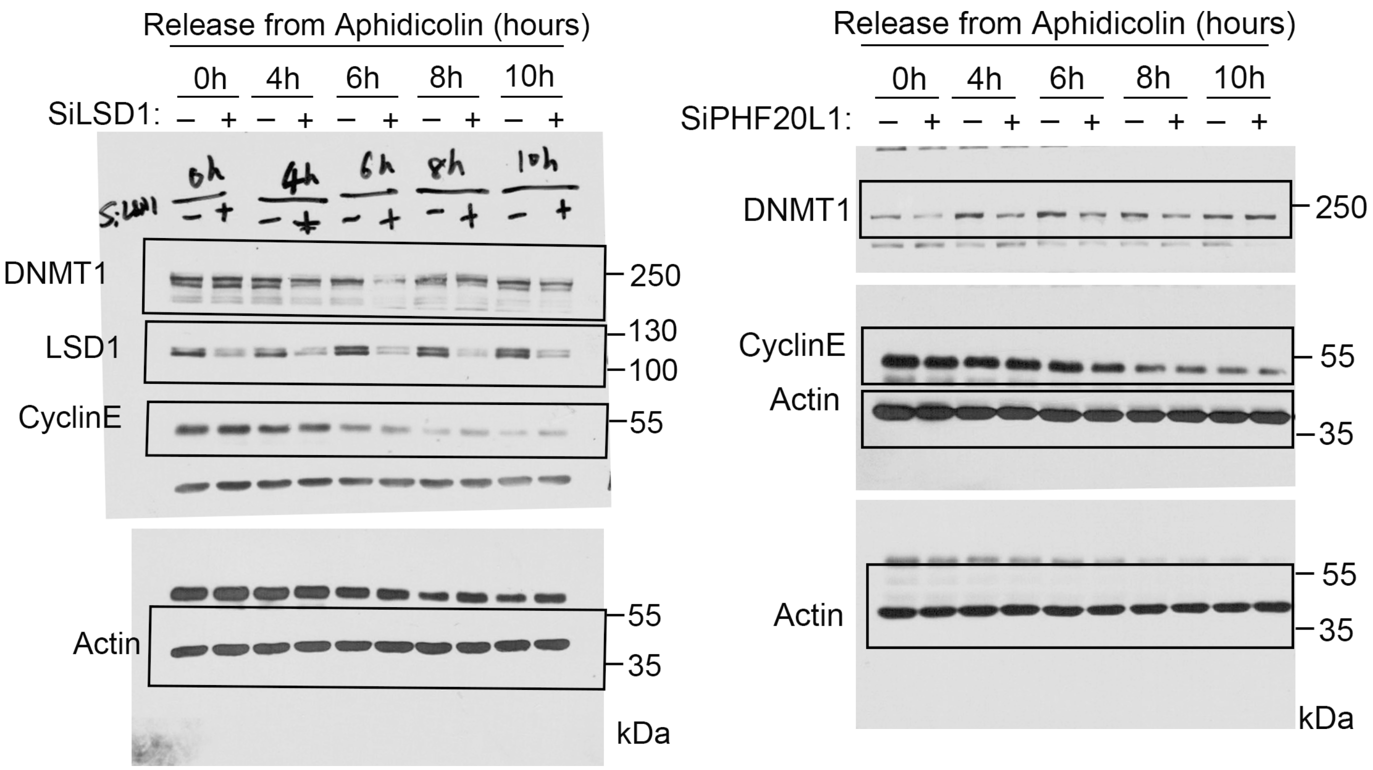

Supplementary Figure 16. Uncropped Images of Western blots from Figure 8E-F
Uncropped Western blots from Fig. 8E(a), Fig. 8F(b).

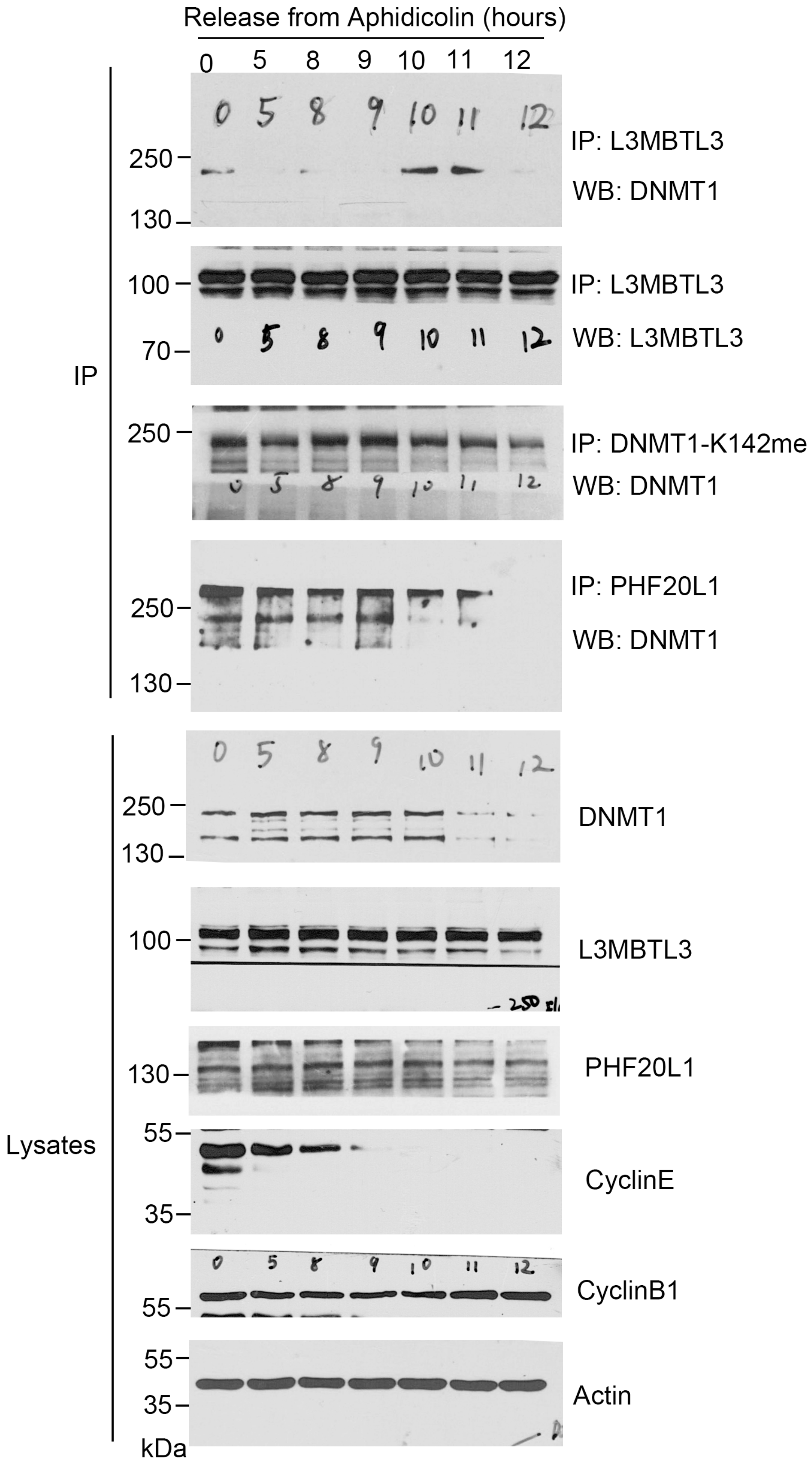

Supplementary Figure 17. Uncropped Images of Western blots from Figure 8G

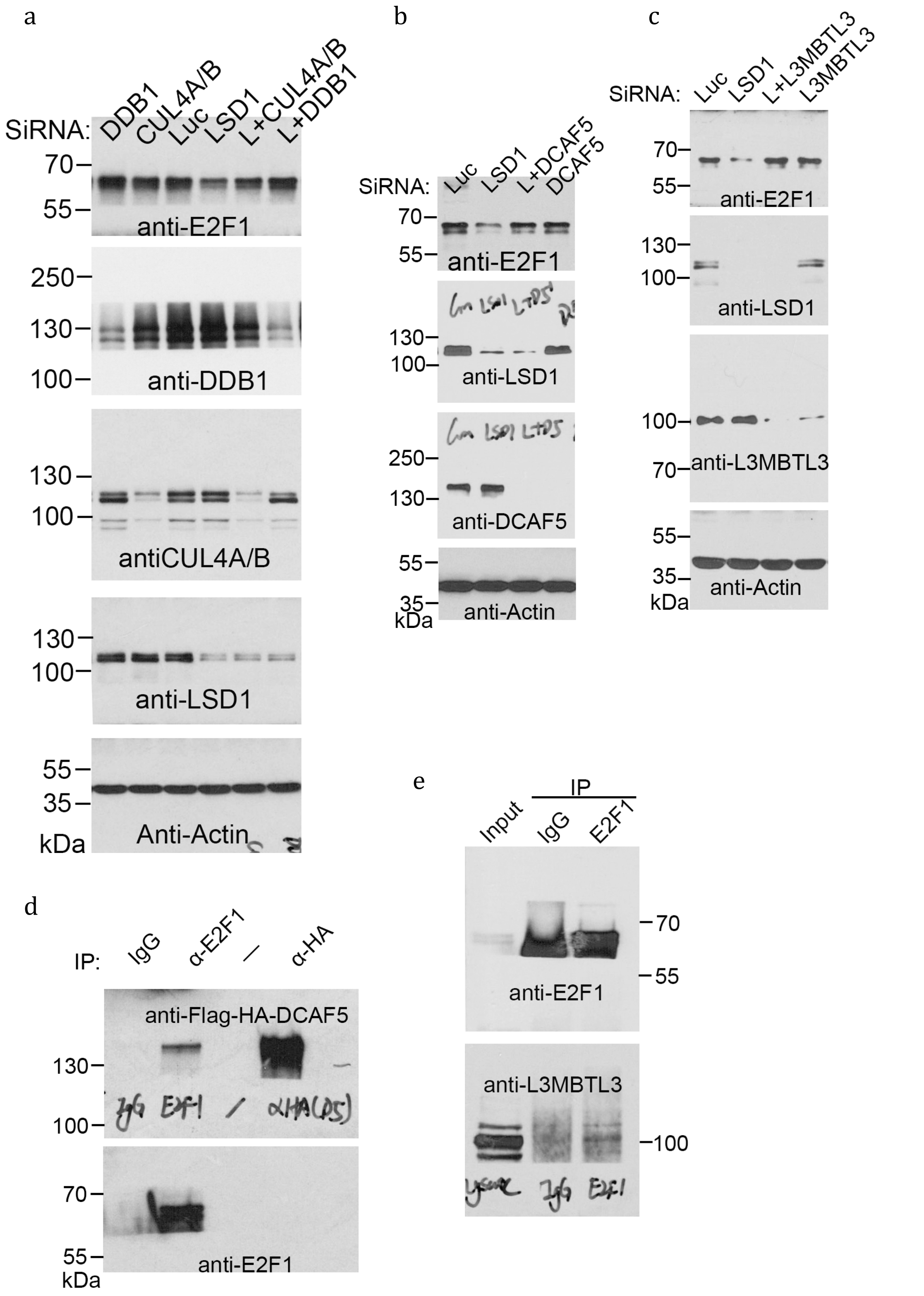

Supplementary Figure 18. Uncropped Images of Western blots from Figure 9A-E
Uncropped Western blots from Fig. 9A(a), Fig. 9B(b), Fig.9C(c), Fig. 9D(d), Fig.9E(e).

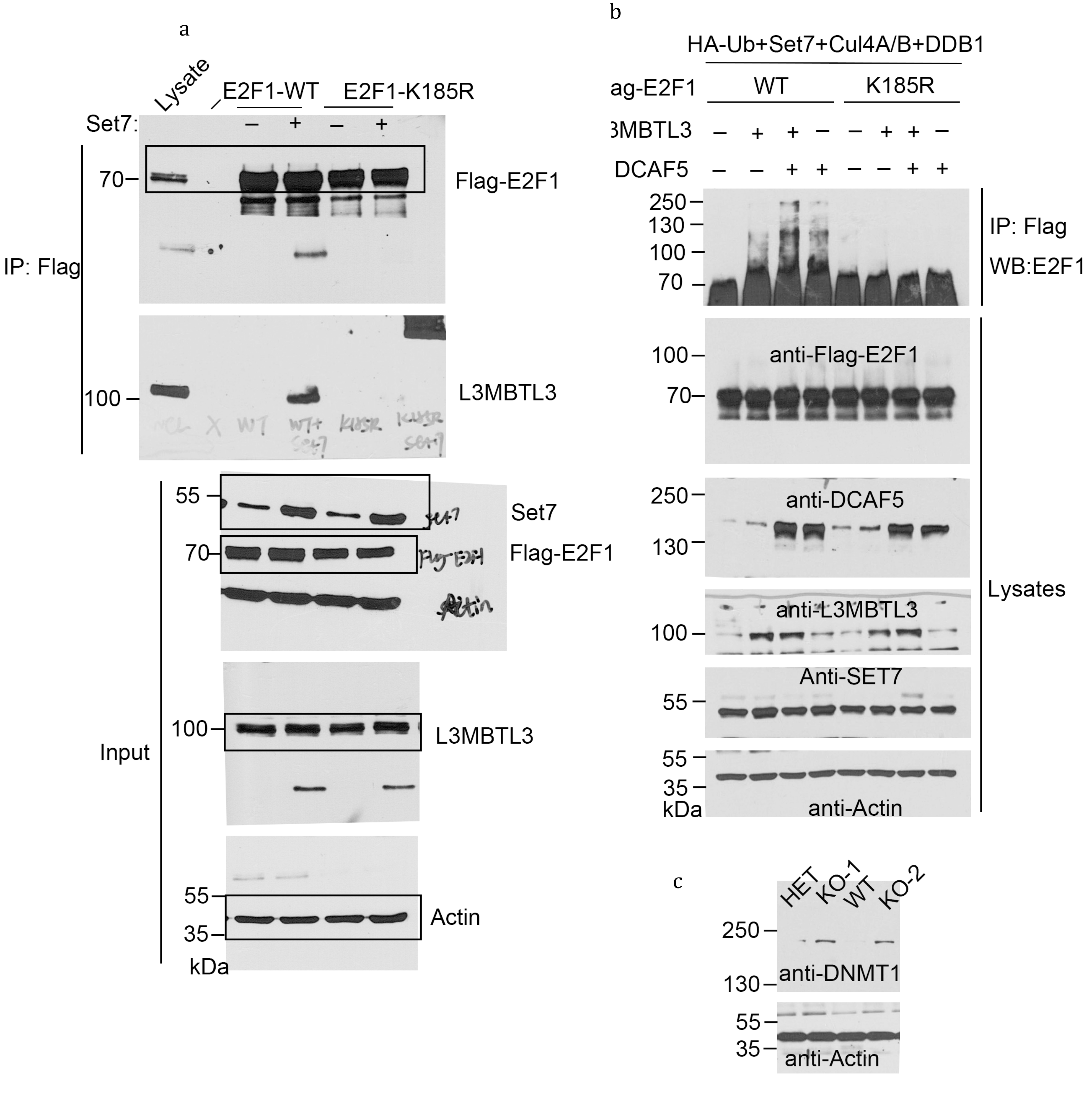

Supplementary Figure 19. Uncropped Images of Western blots from Figure 9F-G and Figure 10B

Uncropped Western blots from Fig. 9F(a), Fig. 9G(b), Fig.10B(c).

Supplementary Table 1: Peptides identified by Mass Spectrometry

Protein Identification	Number of peptides	NCBI Accession Number
DNMT1	186	NM_001130823.1
PHF20L1	13	NM_016018.4
DDB1	8	NM_001923.4
L3MBTL3	7	NM_032438.3
DNMT3A	7	NM_022552.4
DCAF5	6	NM_003861.2
UHRF1	5	NM_001290052.1
UHRFBP1	3	NM_017754.3
DNMT3B	3	NM_175848.1
UHRF2	2	NM_152896.2
UHRFBP1L	2	NM_001006947.1
CUL4B	2	NM_003588.3
CSN2	2	NM_004236.3

The Flag-tagged DNMT1 protein complex was purified by anti-Flag antibody affinity chromatography from HCT116 expressing the Flag tag at the carboxyl terminus of one of the endogenous DNMT alleles. The proteins in the Flag-DNMT1 complexes were fractionated in protein SDS gel. Protein bands were excised, trypsinized, and fractionated on a nano-liter liquid chromatography. The peptides were identified by mass spectrometry analyses in an Orbitrap XL mass spectrometry system with the Protein Discovery software. Three independent mass spectrometry analyses were performed for the identity of a specific peptide/protein in repeated purification samples. Only one preparation sample is shown.
